# Broadband infrared LEDs based on europium-to-terbium charge transfer luminescence

**DOI:** 10.1038/s41467-020-17469-x

**Published:** 2020-07-20

**Authors:** Jonas J. Joos, David Van der Heggen, Lisa I. D. J. Martin, Lucia Amidani, Philippe F. Smet, Zoila Barandiarán, Luis Seijo

**Affiliations:** 1 LumiLab, Department of Solid State Sciences, Ghent University, Krijgslaan 281/S1, 9000 Gent, Belgium; 2 Center for Nano- and Biophotonics (NB Photonics), Ghent University, Technologiepark Zwijnaarde 15, 9052 Gent, Belgium; 3 European Synchrotron Radiation Facility (ESRF), 71 Avenue des Martyrs, 38000 Grenoble, France; 4 Departamento de Química, Facultad de Ciencias, Universidad Autónoma de Madrid, Módulo 13, c/Francisco Tomás y Valiente, Ciudad Universitaria de Cantoblanco, 28049 Madrid, Spain; 5 Instituto Universitario de Ciencia de Materiales Nicolás Cabrera, Universidad Autónoma de Madrid, 28049 Madrid, Spain; 6 Condensed Matter Physics Center (IFIMAC), Universidad Autónoma de Madrid, 28049 Madrid, Spain; 7 Present Address: Helmholtz-Zentrum Dresden-Rossendorf, 01314 Dresden, Germany

**Keywords:** Inorganic LEDs, Electronic properties and materials, Photonic devices

## Abstract

Efficient broadband infrared (IR) light-emitting diodes (LEDs) are needed for emerging applications that exploit near-IR spectroscopy, ranging from hand-held electronics to medicine. Here we report broadband IR luminescence, cooperatively originating from Eu^2+^ and Tb^3+^ dopants in CaS. This peculiar emission overlaps with the red Eu^2+^ emission, ranges up to 1200 nm (full-width-at-half-maximum of 195 nm) and is efficiently excited with visible light. Experimental evidence for metal-to-metal charge transfer (MMCT) luminescence is collected, comprising data from luminescence spectroscopy, microscopy and X-ray spectroscopy. State-of-the-art multiconfigurational ab initio calculations attribute the IR emission to the radiative decay of a metastable MMCT state of a Eu^2+^-Tb^3+^ pair. The calculations explain why no MMCT emission is found in the similar compound SrS:Eu,Tb and are used to anticipate how to fine-tune the characteristics of the MMCT luminescence. Finally, a near-IR LED for versatile spectroscopic use is manufactured based on the MMCT emission.

## Abstract (web summary)

Broadband near-infrared (IR) light-emitting diodes (LEDs) are desirable for smart devices and bio-imaging applications, but the efficiency is limited by the phosphor materials. The authors report broadband emission in Eu-Tb co-doped CaS due to metal-to-metal charge transfer between dopants, and build a broadband near-IR LED with output surpassing the state of the art.

## Introduction

There is an urgent technological demand for broadband infrared (IR) phosphors to build phosphor-converted IR light-emitting diodes (pc-LEDs) with a broad spectral output. Broadband near-IR LEDs are a promising energy-efficient alternative for incandescent lamps for applications that rely on near-IR spectroscopy^1,2^. A large market that is presently explored is IR LEDs for the use in smart devices (e.g., mobile phones) to analyze food: information about its freshness, caloric value, or allergen content can be derived from the built-in spectrometer and IR LED^3^. Proof of concepts are demonstrated, but current state-of-the-art materials do not reach the desired energy efficiency, mostly due to the low absorption strength of the parity-forbidden 3*d*–3*d* transitions of the Cr^3+^ or Mn^2+^ ions that are used^4–10^. Alternatively, near-IR parity-allowed Eu^2+^ 5*d*–4*f* emission has been recently proposed^11^. Higher external quantum efficiencies can thus be achieved; however, at the cost of a smaller IR bandwidth. Material technologies and design strategies to obtain broadband IR phosphors that have a high absorption strength in the visible are hence highly desired to get this technology off the ground.

Other applications follow from the transparency of biological tissue for near-IR light and are hence situated in medicine^12^. This comprises imaging without the use of radioisotopes^13–15^, analyzing tissue during biopsy or endoscopy^16^, and IR therapy^17^. Also here, activators such as Cr^3+^ or Nd^3+^ feature substandard low excitation efficiencies due to the forbidden character of the used 3*d*–3*d* or 4*f*–4*f* transitions^13,15^. Circumventing this limitation can be done by incorporating Eu^2+^ in a suitable host, where the allowed 5*d*–4*f* emission is shifted towards longer wavelengths. A promising host for Eu^2+^ is CaS, where emission ~650 nm was shown in nanoparticles^18,19^. Upon co-doping with Dy^3+^, trapping can be induced upon ex situ UV excitation, after which red emission can be obtained upon in situ IR stimulation. As a downside, the red Eu^2+^ emission in this compound lies only partly in the first optical window of human skin tissue^12^, and would hence benefit from a further shift to the IR.

Large efforts have already been undertaken to optimize IR luminescent materials for the above-mentioned applications, starting from the well-known luminescence transitions such as 3*d*–3*d*, 4*f*–4*f*, or 5*d*–4*f* ^4–10,13,20^. On the contrary, charge transfer (CT) states have remained below the radar because they often cause luminescence quenching, rather than generating luminescence themselves. Nonetheless, the so-called anomalous emission in several phosphors has recently been attributed to intervalence CT (IVCT) transitions^21–26^, that is, electron transfers between two lanthanide dopants that differ only in oxidation state. CT between two different lanthanide elements, that is, metal-to-metal CT (MMCT) states, has not been reported as such. The only exception is the tentative assignment of a direct lanthanide-to-lanthanide CT absorption by Poolton et al.^27^ and concerns a Ce^3+^ + Sm^3+^ → Ce^4+^ + Sm^2+^ MMCT in YPO_4_.

CT states between dopants remain often unnoticed because their absorption bands are very weak, yet they are important^28^. The multivalent nature of lanthanide and transition metal ions induces numerous CT states at low energy, intercalating with the excited states that are typically responsible for the luminescence of the individual ions. Therefore, they are expected to significantly alter the excited state dynamics by quenching existing or by generating new luminescent levels.

Here, broadband IR emission is reported upon addition of Tb to the red phosphor CaS:Eu^2+^. This broadband IR emission can be efficiently pumped with long-wavelength visible light, substantiating an effective improvement of existing IR phosphors for pc-LED applications and for in vivo biomedicine without the need for prior ex situ charging nor expensive detection in the second or third optical window.

CaS:Eu^2+^,Tb^3+^ and SrS:Eu^2+^,Tb^3+^ are investigated in detail in a combined experimental–theoretical study that evidences that the observed IR emission is due to the radiative decay of MMCT states of Eu^2+^–Tb^3+^ pairs, a type of luminescence that was hitherto not described to the best of our knowledge. The ab initio multiconfigurational calculations explain why the MMCT luminescence is found for CaS:Eu^2+^,Tb^3+^, but not for SrS:Eu^2+^,Tb^3+^. This knowledge is utilized to show that MMCT emission can only be obtained when several conditions in terms of the electronic structure of the lanthanide pair, and the structural rigidity of the host crystal are fulfilled. This goes beyond the mere location of energy levels, but comprises vibrational frequencies and lanthanide–ligand bond lengths as well. The reported IR phosphor is finally applied to construct a broadband near-IR LED with a radiant output that surpasses the current state of the art.

## Results and discussion

### Photoluminescence spectra

**Fig. 1:**
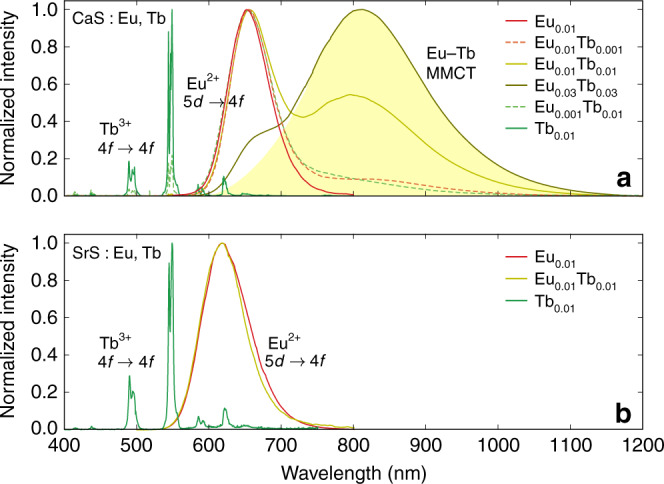
Photoluminescence emission spectra. Photoluminescence emission spectra of Eu^2+^- and Tb^3+^-codoped CaS (**a**) and SrS (**b**) phosphors. The spectra were collected upon 285 nm excitation for the Eu- and Eu/Tb-codoped powders and upon 305 nm excitation for the Tb-doped powders. The spectral shape of the infrared metal-to-metal charge transfer (MMCT) emission is highlighted in yellow as a guide to the eye. All spectra were measured at room temperature.

**Fig. 2:**
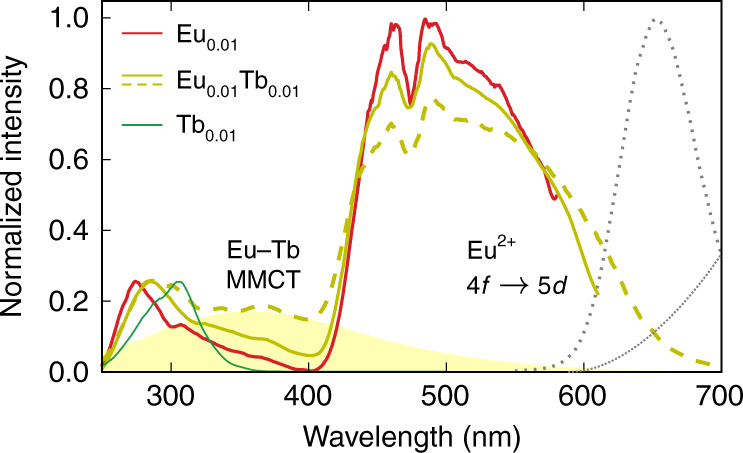
Photoluminescence excitation spectra. Photoluminescence excitation spectra of Eu^2+^- and Tb^3+^-codoped CaS phosphors. Spectra were collected for the Tb^3+^ 4*f*–4*f* emission (545 nm, green solid lines), the Eu^2+^ 5*d*–4*f* emission (650 nm, red and gold solid lines) and the Eu–Tb metal-to-metal charge transfer (MMCT) emission (790 nm, dashed line). The estimated spectral profile of the Eu–Tb MMCT excitation band is highlighted in yellow. The Eu^2+^ 5*d*–4*f* and Eu-Tb MMCT emission spectral profiles are shown in addition (gray dotted lines). All spectra were measured at room temperature.

The photoluminescence (PL) emission spectra for various MS:Eu,Tb (M = Ca,Sr) singly and codoped powders are shown in Fig. 1. Singly Eu-doped MS shows the characteristic broadband emission, peaking ~650 and 615 nm for CaS and SrS, respectively. This band is attributed to the radiative de-excitation of the 4*f*^6^5*d*^1^ states of Eu^2+^ towards the 4*f*^7^(^8^*S*) ground state^29–31^. The associated excitation spectrum as shown in Fig. 2 consists of a very broad band, ranging from 410 to 610 nm, composed of numerous transitions towards the dense $4{f}^{6}5d{t}_{{\rm{2}}g}^{1}$ manifold^31,32^. No trace of Eu^3+^ line emission is found. This does however not exclude its presence, because IVCT states are known to quench the Eu^3+^ emission in case it pairs with Eu^2+^ ions^28^. However, high-energy-resolution fluorescence-detected X-ray absorption near-edge structure (HERFD-XANES^33,34^) spectra show that no Eu^3+^ is present in the prepared samples within the detection limits (see Supplementary Fig. 2).

Singly Tb-doped MS shows characteristic Tb^3 + ^^5^*D*_4_ → ^7^*F*_*J*_ line emission across the visible range (green curve in Fig. 1), most notably in the green (545 nm, *J* = 5). This intraconfigurational 4*f*^8^ emission can be excited by a relatively high-lying 4*f*^8^ → 4*f*^7^5*d*^1^ excitation band in the near-UV (Fig. 2)^35–37^. In case of CaS and SrS, the fundamental absorption of the host lies in the same energy range, and the near-UV absorption and excitation bands are likely the result of a mixture of host- and dopant-related transitions^30,31^.

When Eu and Tb are combined in the same sample, an additional IR emission band emerges in case of CaS, but not in case of SrS. This band is highlighted in Fig. 1. It peaks at 810 nm (12,345 cm^−1^) and is very broad, with a full-width at half-maximum of 195 nm (2960 cm^−1^). On the high-energy side, it overlaps with the Eu^2+^4*f*^6^5*d*^1^ → 4*f*^7^ luminescence around 650 nm and extends up to 1100–1200 nm. Its excitation spectrum (dashed curve in Fig. 2) is very similar as for the Eu^2+^ luminescence, where the $4{f}^{7}\to 4{f}^{6}5d{t}_{{\rm{2}}g}^{1}$ band can be identified, with a small redshift of 20 nm, corresponding to about 500 cm^−1^. In addition to this band, some excitation intensity can be found in the region around 370 nm where no allowed transitions for Eu^2+^ are found^31^, suggesting the presence of additional excited states when Eu and Tb are codoped. These features are also visible at low temperature, as well as in the diffuse reflectance spectra (see Supplementary Figs. 6 and 7).

Lifetime measurements (see Supplementary Fig. 9 and Supplementary Table 1) indicate that the IR emission bands feature a very similar decay behavior than the Eu^2+^ 5*d* → 4*f* emission, with time constants around 500 ns at room temperature^38^.

In order to exclude that this previously unseen emission band is due to the used precursors, codoped powders from different precursor batches were prepared, using fluorides, oxides, and sulfides as lanthanide precursors^39–42^. All syntheses where Eu and Tb were both present as dopants resulted in the same IR emission band, while this IR band was always absent in case that only one dopant was used (see Supplementary Fig. 4) . The similarity of the IR emission regardless of the synthesis conditions suggests that the details of the charge compensation mechanism for Tb^3+^ do not directly affect the IR emission. Explicit compensation of Tb^3+^ by adding a monovalent codopant such as Na^+^ is shown to be detrimental for the luminescence properties, leading to a efficiency drop of a factor 10 (see Supplementary Fig. 8). The reason is that, when Tb^3+^ is extrinsically compensated, the formation of Eu^3+^ will also be favored^29^, generating undesired Eu^2+^–Eu^3+^(–Na^+^) centers in addition to the intended Eu^2+^–Tb^3+^(–Na^+^) centers. The former centers are responsible for the luminescence efficiency drop by IVCT quenching^28,43^.

This check, along with the demonstrated phase purity (from X-ray diffraction (XRD), see Supplementary Fig. 1) and the presence of only one oxidation state for both dopants in the absence of extrinsic charge compensation, that is, Eu^2+^ and Tb^3+^ (from HERFD-XANES, see Supplementary Fig. 2) strongly suggests that this IR emission band is a physical effect that emerges due to an interaction between the Eu^2+^ and Tb^3+^ centers in the calcium sulfide crystal.

To acquire more information about this peculiar luminescence, its properties are investigated as a function of the Eu and Tb doping concentrations. The PL emission spectra indicate that the Tb^3+^ intraconfigurational 4*f*^8^ emission is firmly diminished upon the addition of Eu^2+^ ions until it completely vanishes (Fig. 1). This is not surprising due to the large overlap between the Tb^3+^ emission and Eu^2+^ excitation spectra, enabling an efficient energy transfer where the Tb^3+^ ion sensitizes the Eu^2+^ luminescence^44,45^. When a small amount of Eu^2+^ is added to a Tb^3+^-doped sample (CaS:Eu_0.001_Tb_0.01_), or vice versa (CaS:Eu_0.01_Tb_0.001_), the IR emission shows up, but with a limited intensity. The relative intensities are comparable in both cases (see Fig. 1). For higher concentrations, 1% for both dopants (CaS:Eu_0.01_Tb_0.01_), the IR emission stands out, featuring a larger integrated intensity than the Eu^2+^5*d* → 4*f* emission. Upon increasing the doping concentrations even more (CaS:Eu_0.03_Tb_0.03_), the IR emission dominates the entire emission spectrum.

The emergence of the IR emission upon Tb addition to CaS:Eu is accompanied by a decrease in PL quantum efficiency (QE), which decreases from 35% for CaS:Eu_0.01_ to 10% for CaS:Eu_0.01_Tb_0.01_ (see Supplementary Fig. 8). This relatively low internal QE is partly compensated by the efficient excitability of the IR emission (see Fig. 2), where >90% of the incident visible (400–600 m) light is absorbed (see Supplementary Fig. 7). It is hence clear that practical applications require a trade-off between conversion efficiency and the fraction of IR in the emission spectrum (see further)^46^.

### Concentration dependence

The concentration-dependent PL study indicates that the intensity of the IR emission scales with the product of the concentrations of both dopants; however, drawing quantitative conclusions is hindered by the limited number of concentrations that can be prepared and does not account for uncontrollable microscopic concentration differences^47–50^. To get a more detailed picture, a microscopic study is performed.

For this, two grains with extreme doping inhomogeneity are explored^51–53^. It should be stressed that these grains were selected for this purpose and that they do not represent the global doping homogeneity of the phosphors, which is much better. As shown in Supplementary Fig. 3 and the accompanying discussion, variations in local concentrations are limited to less than a percent, corresponding to a decent doping homogeneity.

**Fig. 3:**
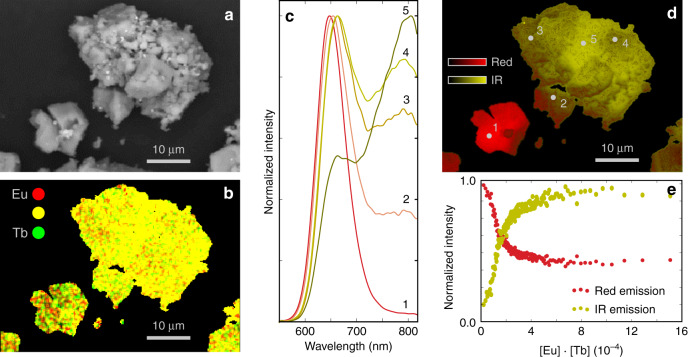
Microscopic SEM-EDX-CL study. Scanning electron microscope (SEM) study on a CaS:Eu_0.01_Tb_0.01_ powder. Backscattered electron image (**a**), along with energy-dispersive X-ray (EDX) maps for terbium and europium (**b**). The colors encode the local terbium (green) and europium (red) concentrations. Simultaneous detection leads to a yellow color. **c** Cathodoluminescence (CL) spectra for a few selected points that are indicated in **d**. **d** False color image, displaying the total integrated CL intensity. **e** shows how the relative contributions of the red Eu^2+^ (integrated from 600 to 650 m) and IR (integrated from 760 to 815 m) luminescence band to the total CL spectrum evolve as a function of the local Eu and Tb concentrations, [Eu] ⋅ [Tb].

The microscopic study of the inhomogeneous grains is shown in Fig. 3. One grain exhibits predominantly red emission (lower left), while the other grain shows a strong IR emission (upper right). From the elemental analysis by energy-dispersive X-ray spectroscopy (EDX), it is clear that the doping is indeed inhomogeneous and that the local Eu and Tb concentrations range from 0 to roughly 4%^54^. As a direct consequence, the cathodoluminescence (CL) spectrum shows strong variations across the sample because the intensity of the IR emission depends strongly on the Eu and Tb concentrations. As an illustration, five local spectra are shown in Fig. 3c. It is clear that the smaller grain on the bottom of the image shows negligible IR emission (Fig. 3c, d), which is compatible with the elemental analysis that suggests that Eu and Tb are well separated in this grain, indicated by a limited amount of yellow in Fig. 3b. For the larger grain, Eu and Tb clearly congregate (there is more yellow in Fig. 3b) and intense IR emission is found (Fig. 3c, d).

From the PL study, it is clear that both Eu and Tb are required to induce IR emission; therefore, the product of both local concentrations, [Eu] ⋅ [Tb], determined per pixel in the scanning electron microscope (SEM)-EDX map, is used as dependent variable to correlate the luminescence properties to the CL spectrum, measured for the same pixel^49,55^. The red and IR contributions to the local CL spectrum are integrated and subsequently analyzed as a function of [Eu] ⋅ [Tb]. Figure 3e was obtained by averaging data points along the abscissa. At low [Eu] ⋅ [Tb] values, the IR emission increases linearly as a function of [Eu] ⋅ [Tb], while the red Eu^2+^ emission decreases accordingly. After the linear increase/decrease, the relative intensities stabilize and the spectrum does not change appreciably upon increasing the product of the doping concentrations above roughly 3 × 10^−4^, which corresponds to a symmetric doping concentration, $\sqrt{[{\rm{Eu}}]\cdot [{\rm{Tb}}]}$, of 1.7%. No spot is found where the red Eu^2+^ emission completely disappears, not in the area displayed in Fig. 3, nor in any other grain that was investigated, nor in the emission spectra of phosphors with doping concentrations >5% (see Supplementary Fig. 5). This equilibrium between red and IR emissions implies that the IR emission is presumably not the result of an energy transfer from Eu^2+^ to another emitting center because it would then be expected for the Eu^2+^ emission to vanish completely if the concentrations are stretched sufficiently^44,56^. Yet, the Eu^2+^ absorption bands are clearly present in the excitation spectrum of the IR band. This means that the IR emission stems directly from a Eu^2+^-containing defect cluster.

### Temperature dependence

**Fig. 4:**
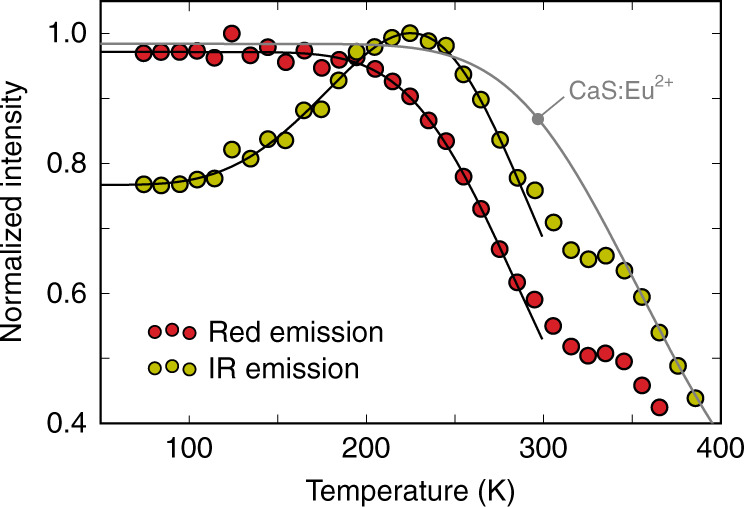
Thermal quenching profiles. Thermal quenching curves for the red and infrared (IR) emission bands of CaS:Eu_0.01_Tb_0.01_, measured upon 470 nm excitation. The black curves are the result of fitting Eqs. (1) and (2) for the red and IR emissions, respectively. The small shoulder ~340 K is likely due to thermoluminescence (TL) and is not explored further here. The gray curve shows the thermal quenching of singly doped CaS:Eu_0.01_ for comparison.

Measurement of the PL intensities as a function of temperature, that is, a thermal quenching (TQ) experiment can reveal more information about the equilibrium between the red and IR emissions. The result is shown in Fig. 4. The red Eu^2+^ emission follows a rather standard behavior, which resembles the shape of a single-barrier model^57^,

1 $${I}_{{\rm{red}}}(T)=\frac{{I}_{0}}{1+A\exp \frac{-\Delta {E}_{T,{\rm{red}}}}{{k}_{{\rm{B}}}T}}.$$

Using this phenomenological model to fit the data yields a barrier height of Δ*E*_*T*,red_ = 1484 cm^−1^ (*A* = 1.07 × 10^3^). This TQ performance is comparable to the one of singly doped CaS:Eu_0.01_ phosphors, which contain sufficient Eu for concentration quenching to be noticeable^29,58,59^.

In contrast to the red emission, which shows the expected TQ behavior, the IR emission looks more complicated as a function of temperature, with an increase in intensity between 100 and 225 K. This indicates that the IR emission is to some extent thermally activated. However, a substantial fraction of the IR emission, ~77% of the maximal output at 225 K, is also being emitted at low temperature. This is reminiscent of the temperature dependence of internal conversion (IC) and inter-system crossing (ISC) in molecular chromophores where rate constants are typically written as the sum of a temperature-dependent and temperature-independent term^60,61^. The IR TQ curve can hence be modeled by combining the ISC rate constant with a single-barrier model for the TQ behavior,

2 $${I}_{{\rm{IR}}}(T)=\frac{{I}_{1}+{I}_{2}\exp \frac{-\Delta {E}_{{\rm{ISC}}}}{{k}_{{\rm{B}}}T}}{1+A\exp \frac{-\Delta {E}_{T,{\rm{IR}}}}{{k}_{{\rm{B}}}T}},$$

where *I*_1_ and *I*_2_ represent the temperature-independent and temperature-dependent rate constants, respectively. Δ*E*_ISC_ is the associated barrier height for the latter. Fitting Eq. (2) to the TQ profile of the IR emission yields Δ*E*_ISC_ = 476 cm^−1^ and Δ*E*_*T*,IR_ = 1500 cm^−1^ as barriers (*I*_1_/*I*_2_ = 9.5 and *A* = 1.61 × 10^3^). The TQ at higher temperature is roughly the same as for the red emission, indicated by the similar energy barriers for quenching.

The intensities of the red and IR emission of the Eu,Tb-codoped phosphor already decrease at lower temperature with respect to the red emission of singly Eu-doped CaS (see gray curve in Fig. 4), featuring Δ*E*_*T*,red_ = 1989 cm^−1^ (*A* = 2.04 × 10^3^, Eq. (1)). This indicates that the addition of Tb opens an additional non-radiative decay channel that is active around room temperature. At higher temperatures, the TQ of the singly Eu-doped and Eu,Tb-codoped phosphors coincide, reaching 50% of the initial intensity at *T*_0.5_ ≈ 375 K. This value is in correspondence with prior studies^58,59,62^, even though values of 475 K have been reported for single crystals^58^, suggesting that efficiency gains are still feasible by optimizing the synthesis.

As shown by a model calculation by Struck and Fonger and subsequent surveys of experimental literature by various authors, the physical meaning of the above-determined energy barriers is rather limited due to tunneling effects and the importance of the details of the electron-vibrational structure on the non-radiative transition probabilities^63–66^. For that reason, Eqs. (1) and (2) should be regarded as strictly empirical prescriptions.

Qualitative interpretation of the above analysis suggests that an excited Eu^2+^ ion in the Eu^2+^, Tb^3+^-codoped material has two radiative decay possibilities, the standard red luminescence, and the IR luminescence which is achieved after some kind of internal transition towards another energy level. If both emissions would originate from the same initial level and differ in final level, the temperature-induced intensification of the IR emission would not be expected.

### MMCT model

**Fig. 5:**
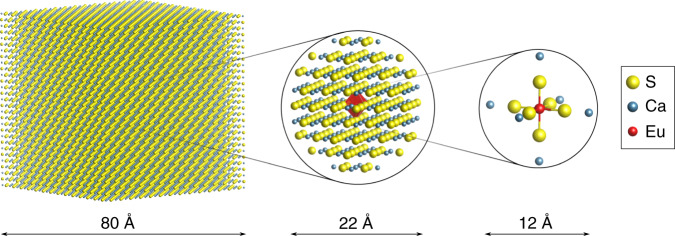
Embedded cluster calculations. Graphical representation of the simulated system and how it is divided in the (LnS_6_M_6_)^2+/3+^ (M = Ca, Sr and Ln = Eu, Tb) cluster, which is treated at the highest level of theory (right), subject to the embedding potential composed of the full-ion ab initio model potentials (AIMP) of the 316 ions in the next four coordination shells (middle) and 24,389 point charges at the crystal lattice sites (left). The indicated distances are for CaS and are slightly larger for SrS.

The experimental findings suggest that MMCT states might be involved in the complicated luminescence of this material. Given the oxidation states of the dopants in this compound, Eu^2+^ and Tb^3+^, the most probable scenario to be investigated are the Eu^3+^-Tb^2+^ MMCT states, that is, those where an electron is transferred from Eu^2+^ to Tb^3+^. To this means, ab initio embedded cluster calculations are employed (see Fig. 5).

Diabatic potential energy surfaces and configurational diagrams of Eu^2+^-Tb^3+^ pairs are obtained from the results of independent embedded cluster calculations^23,67^. This approach has proven its reliability by explaining the anomalous emission of several Ce- and Yb-doped phosphors^23,25,26,68^, predicting the existence of absorption bands due to IVCT states in Eu-doped phosphors^28^ and by showing the role of MMCT states at quenching luminescent levels of Pr^3+^^69^.

The configurational coordinate diagrams along the breathing mode for Eu^2+^, Tb^3+^, Eu^3+^, and Tb^2+^ are the ingredients for the electron transfer diagrams that describe the Eu-to-Tb MMCT transitions according to the recipe in refs. ^22,23^. Calculations are performed for CaS and SrS hosts. Details, intermediate and final results are collected in Supplementary Tables 2–7 and in Supplementary Figs. 10–15.

The excited state landscape of Eu^2+^ was previously discussed in detail in ref. ^31^ and is practically defined by a single ground state level, 4*f*^7^(^8^*S*_7/2_), separated from a very dense 4*f*^6^5*d*^1^ manifold by ~16,000–17,000 cm^−1^. Eu^3+^ and Tb^3+^ feature conjugate ground state configurations with 4*f*^6^(^7^*F*_0_) and 4*f*^8^(^7^*F*_6_) multiplets, respectively. The higher-lying 4*f*^6^(^5^*D*_0_) and 4*f*^8^(^5^*D*_4_) states are the main 4*f*–4*f* emitting levels. The 4*f*^*N* − 1^5*d*^1^ configurations feature high excitation energies for these trivalent lanthanides and are hence unnecessary for the current calculation. For Tb^2+^, the 4*f*^8^5*d*^1^ and 4*f*^9^ configurations are close in energy, the former constituting the ground state in CaS (1Γ_7*g*_), while a reversed order is found for SrS (1*Γ*_7*u*_). More details about the electronic structures of Tb^3+^ and Tb^2+^ in CaS and SrS are given in the Supplementary Discussion, in particular regarding the relative energy of the 4*f*^8^5*d*^1^ and 4*f*^9^ manifolds of Tb^2+^. The equilibrium Eu–S and Tb–S bond lengths and breathing mode vibrational frequencies are summarized in Table 1. Prior comparisons with experimental results for Eu^2+^ and Eu^3+^ proved an excellent quantitative agreement of <300 cm^−1^ for excitation energies and at most a few % for equilibrium bond lengths and vibrational frequencies^31^.

**Table 1:** Calculated spectroscopic parameters.

	CaS	SrS
Eu–S equilibrium distances		
Eu^2+^(^8^*S*_7/2_)	2.876	2.974
Eu^3+^(1*A*_1*g*_)	2.729	2.800
Difference	0.147	0.174
Tb–S equilibrium distances		
Tb^3+^(1*A*_1*g*_)	2.708	2.780
Tb^2+^(1*Γ*_7*g*/*u*_)	2.833	2.948
Difference	−0.126	−0.169
LnS_6_ breathing mode		
Vibrational frequencies		
Eu^2+^(^8^*S*_7/2_)	286	273
Tb^3+^(1*A*_1*g*_)	306	284
Mean values	296	279
Eu^3+^(1*A*_1*g*_)	307	286
Tb^2+^(1*Γ*_7*g*/*u*_)	277	233
Mean values	292	242
MMCT properties		
Equilibrium energy	19,968	20,403
Equilibrium *Q*_et_	0.428	0.548
Diabatic energy barriers for hopping		
Eu^2+^ Tb^3+^ ↔ Eu^3+^ Tb^2+^		
1*Γ*_8*g*_ 1*A*_1*g*_ → 1*A*_1*g*_ 1*Γ*_7*g*/*u*_	3406	3742
1*Γ*_8*g*_ 1*A*_1*g*_ ← 1*A*_1*g*_ 1*Γ*_7*g*/*u*_	748	1602
Diabatic energy barriers for Non-radiative decay MMCT		
Eu^3+^ Tb^2+^ ↔ Eu^2+^ Tb^3+^		
1*A*_1*g*_ 1*Γ*_7*g*/*u*_ → ^8^*S*_7/2_ 3*A*_1*g*_	786	94

Vertical electronic transition energies, diabatic energy barriers, and distance from the configurational coordinate diagrams. *Γ*_7*g*_ and *Γ*_7*u*_ are the lowest levels of Tb^2+^ in CaS and SrS, respectively. Energies and vibrational frequencies in cm^−1^; distances in Å.

Within the diabatic approximation, the energy level scheme of a lanthanide pair can be constructed by combining all the levels of the individual ions, the resulting energy is given by addition of the individual energies with the Coulomb and exchange energies between the two lanthanides. The last two contributions are assumed to be state independent^67^. The resulting energy levels are uniquely labeled by combining both individual labels. As an example, the ground state of a Eu^2+^–Tb^3+^ pair is denoted as 4*f*^7^(^8^*S*_7/2_)–4*f*^8^(1*A*_1*g*_). Diabatic potential energy curves have proven their use by successfully explaining qualitative trends of CT processes upon chemical substitutions in host compounds^22,23,28,43^.

**Fig. 6:**
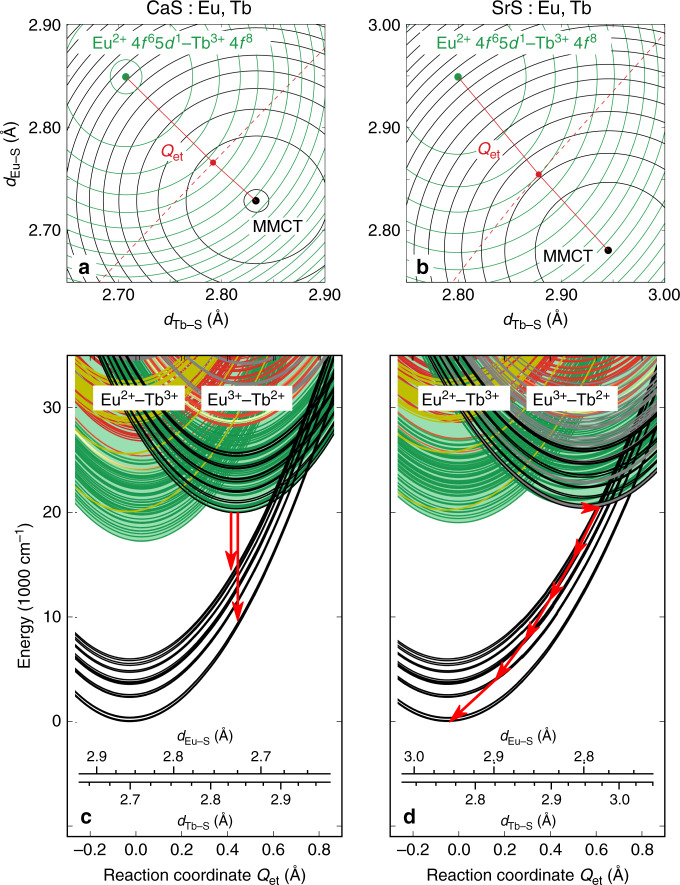
Metal-to-metal charge transfer many-electron diagrams. Contour plots of selected potential energy surfaces in the two-dimensional configurational space (*d*_Eu–S_, *d*_Tb–S_) (**a**, **b**) and configurational coordinate diagrams along the electron transfer reaction coordinate (*Q*_et_) (**c**, **d**) for Eu–Tb pairs in CaS (**a**, **c**) and SrS (**b**, **d**). The configurational coordinate diagrams display the following levels for the Eu^2+^–Tb^3+^ configuration: 4*f*^7^(^8^*S*)–4*f*^8^(^7^*F*) (black), $4{f}^{6}5d{t}_{{\rm{2}}g}^{1}-4{f}^{8}(1\,{A}_{{\rm{1}}g})$ (green for high-spin states, red for low-spin states) and 4*f*^7^(^8^*S*)–4*f*^8^(^5^*D*, *L*, *G*) (gold), and following levels for the Eu^3+^–Tb^2+^ configuration: 4*f*^6^(^7^*F*)–4*f*^8^5*d*^1^(1*Γ*_7*g*_) (black), 4*f*^6^(1*A*_1*g*_)–4*f*^8^5*dt*_2*g*_ (green for high-spin states, red for low-spin states), and 4*f*^6^(1*A*_1*g*_)–4*f*^9^ (gray). The quasi-continuum formed by all other levels of the Eu–Tb pair, where none of both ions is in its ground state are represented by the green-colored background. The contour plots display the isolines corresponding to the Eu^2+^4*f*^6^5*d*^1^(1*Γ*_8*g*_)–Tb^3+^4*f*^8^(1*A*_1*g*_) and Eu^3+^4*f*^6^(^7^*F*)–Tb^2+^4*f*^8^5*d*^1^(1*Γ*_7*g*_) (lowest metal-to-metal charge transfer (MMCT) state) surfaces. The reaction coordinate is indicated in red, along with the intersection between both surfaces (dashed red line).

For every energy level of the Eu–Tb pair, a two-dimensional potential energy surface is obtained, spanned by the breathing modes of the EuS_6_ and TbS_6_ moieties. Every point in this two-dimensional space hence corresponds to a unique pair of (*d*_Tb−S_,*d*_Eu−S_). The equilibrium point of the Eu^2+^–Tb^3+^ states correspond to a relatively large *d*_Eu−S_ value (divalent ion) and relatively small *d*_Tb−S_ value (trivalent ion). The Eu^2+^–Tb^3+^4*f*^6^5*d*^1^(1*Γ*_8*g*_)–4*f*^8^(1*A*_1*g*_) potential energy surface is represented in the top panels of Fig. 6 for CaS and SrS (green contours), along with the lowest MMCT level, Eu^3+^–Tb^2+^4*f*^6^(1*A*_1*g*_)–4*f*^8^(1*Γ*_7g_) (black contours), where *d*_Eu−S_ decreased (trivalent ion) and *d*_Tb−S_ increased (divalent ion). The intersection of both potential energy surfaces is a curved line (dashed red line in the contour plots of Fig. 6).

The lower panels of Fig. 6 display the configurational coordinate diagrams for Eu^2+^–Tb^3+^ pairs in both host compounds along the electron transfer reaction coordinate, *Q*_et_. This coordinate is shown in the contour plots and is defined here as the piecewise straight line that connects the equilibria of the Eu^2+^–Tb^3+^4*f*^6^5*d*^1^(1*Γ*_8*g*_)−4*f*^8^(1*A*_1*g*_) and Eu^3+^–Tb^2+^4*f*^6^(1*A*_1*g*_)−4*f*^8^5*d*^1^(1*Γ*_7g_) potential energy surfaces in the two-dimensional (*d*_Eu−S_, *d*_Tb−S_) configurational space with their saddle point, that is, the minimum of the intersection of both surfaces. This one-dimensional diagram is a simplification of the two-dimensional space that is probed for convenient visualization. Reported data such as location of minima, crossing points, barriers, and transition energies are however obtained from the two-dimensional surfaces.

The configurational coordinate diagrams show that the Eu^3+^–Tb^2+^ MMCT configuration gives rise to low-lying states, lying around 20,000 cm^−1^ above the ground state (Table 1). The presence of these MMCT states alters the excited state dynamics after 4*f*^7^ → 4*f*^6^5*d*^1^ excitation of the Eu^2+^ ion. The excited electron can be non-radiatively transferred to the Tb^3+^ ion, forming a transient Eu^3+^–Tb^2+^ pair. Shortly, the Tb^2+^5*d* electron is transferred back to the Eu^3+^4*f* orbital, leading to a decay of the MMCT state.

Different decay channels are found in the case of Eu,Tb-codoped CaS or SrS, evoked by their structural differences. In case of CaS, the minimum of the lowest MMCT state (4*f*^6^(1*A*_1*g*_)−4*f*^8^5*d*^1^(1*Γ*_7*g*_)) is metastable. Therefore, radiative decay of this state can be expected to the structurally stressed 4*f*^7^(^8^*S*_7/2_)−4*f*^8^(^6^*F*_*J*_) ground state (red arrows in Fig. 6c). In SrS, the minimum of the lowest MMCT state is crossed by the branches of the stressed 4*f*^7^(^8^*S*_7/2_)−4*f*^8^(^6^*F*_*J*_) states, enabling efficient non-radiative decay due to fast bottom crossover (red arrows in Fig. 6d) that impedes any radiative decay. The associated diabatic energy barrier is 94 cm^−1^, and will likely disappear or be of negligible size in an adiabatic calculation. This is indeed the behavior, which is experimentally found for Eu,Tb-codoped CaS and SrS.

Due to the large horizontal offset between ground and MMCT states, the resulting emission band in case of CaS:Eu,Tb is expected to be broad. The MMCT emission is predicted to start around 11,000 cm^−1^ (900 nm), which is at slightly lower energy than the experimental IR emission, which starts ~14,000–15,000 cm^−1^. This quantitative discrepancy between experimental and computed transition energies is in line with what can be expected from the diabatic approximation^23,67^.

The low-lying MMCT states do not only cause an additional emission band. Vertical excitation from the ground state towards the MMCT states is possible starting from about 28,000 cm^−1^ (360 nm), which coincides with the energy range where the low-spin $4{f}^{6}5d{t}_{{\rm{2}}g}^{1}$ levels are found. The latter are spectroscopically invisible by direct excitation from the 4*f*^7^(^8^*S*_7/2_) ground state because of the spin selection rule^31^. The presence of the Eu–Tb pairs and the MMCT states induces transition probability in this otherwise forbidden energy region as evidenced by the excitation band ~370 nm in the experimental spectrum (Fig. 2). An estimated spectral shape is highlighted as a guide to the eye. This MMCT absorption is also visible in the excitation spectrum of the regular Eu^2+^ red emission at 650 nm for the CaS:Eu_0.01_,Tb_0.01_ sample, indicating that a substantial fraction of the dopants can already interact in the studied concentration range.

The PL excitation spectrum of the MMCT emission (dashed line in Fig 2) indicates that it can be excited with the same wavelengths as the regular Eu^2+^ emission, even when the photon energy of the excitation light is insufficient to reach an MMCT branch by vertical excitation from the ground state. This can be explained by considering the double-well shape of the potential energy surface that is formed by the Eu^2+^(1*Γ*_8*g*_)–Tb^3+^(1*A*_1*g*_) red-emitting and the Eu^3+^(1*A*_1*g*_)–Tb^2+^(1*Γ*_7g_) IR-emitting MMCT state. Even at low temperature, when the barrier cannot be thermally overcome, quantum mechanical tunneling will partially populate the MMCT state. This does not only explain why both emissions always appear together, but also why the IR emission is intensified when sufficient thermal energy is available to overcome the barrier and why the luminescent lifetimes for both emission bands are comparable.

### Broadband IR LED

The broadband IR MMCT emission is now applied to construct an IR pc-LED that can directly be used for the numerous above-mentioned spectroscopic applications. Because of the high absorption strength of the parity-allowed 4*f*−5*d* transitions of Eu^2+^, higher external quantum efficiencies can be achieved than with the current state-of-the-art, Cr^3+^-based phosphors^4–6,8–10^. Furthermore, the extremely broad MMCT emission extends the covered spectral range by several hundreds of nanometers to the IR compared to red/near-IR single Eu^2+^5*d*−4*f* emission^11^.

**Fig. 7:**
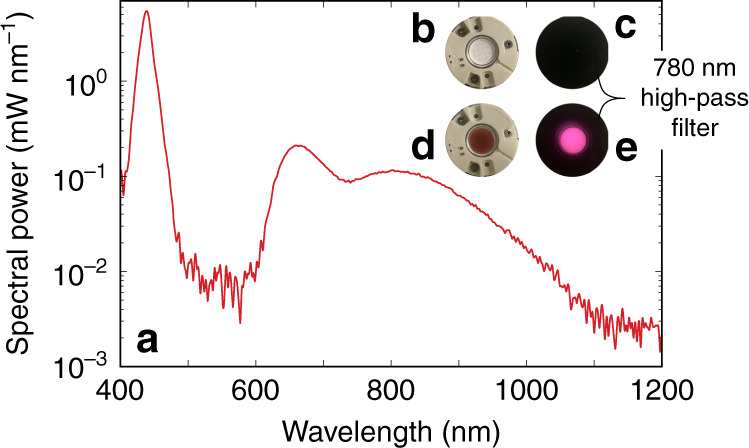
Broadband near-IR LED. **a** Spectral output of the constructed broadband infrared (IR) light-emitting diode (LED). The inset shows the LED design, comprising a blue pumping LED (**b**), combined with the CaS:Eu,Tb broadband infrared phosphor (**d**), under operation below a 780-nm high-pass cut-off filter without (**c**) and with (**e**) the infrared phosphor present, captured by a camera with a near-IR responsivity.

  Figure 7 displays the spectrum, expressed in mW nm^−1^, for the obtained LED, where a blue 450 nm pumping LED was used. The LED features a width of 430 nm in the IR, ranging from 620 to 1050 nm. The operation of the IR LED is illustrated by the pictures in Fig. 7b–e. Here, a high-pass cut-off filter of 780 nm is used to filter out the transmitted blue pumping light in order to appreciate the brightness of the IR emission. The pictures were taken with a camera that is also sensitive to near-IR light, as the emission is barely visible by the naked eye. The total radiant flux of the IR part of the emission amounts to 38 mW. This value surpasses the current Cr^3+^-based state-of-the-art, having IR radiant fluxes in the range of 20–25  mW^4–6,8–10^, by 50%.

It is clear that this broadband IR MMCT emission has a huge application potential. The next step comprises further optimization of its luminescence to arrive at higher efficiencies and to enable some spectral tuning to optimally meet the requirements of the different applications. As the luminescence mechanism in CaS:Eu,Tb was resolved in detail by our ab initio calculations, some prospects and further insights can be given based on these findings.

As shown, SrS is not a suitable host to obtain a luminescent MMCT state. There are two main differences between CaS and SrS that are crucial in this regard. First, the vibrational frequency is smaller by a few tens of cm^−1^ in case of SrS (242 cm^−1^, compared to 292 cm^−1^ for CaS, see Table 1), which causes a slight opening of the branches that cross the MMCT state. This effect is not large because the vibrational frequency differs only by a small amount between CaS and SrS. Second, the offset in equilibrium geometry for the ground and MMCT states is larger for SrS (*Q*_et_ = 0.548 Å, compared to 0.428 Å for CaS). This is the direct consequence of the larger bond length difference between divalent and trivalent lanthanides with respect to CaS (see Table 1) and causes the MMCT minimum to be pushed into the 4*f*^7^(^8^*S*_7/2_)−4*f*^8^(^6^*F*_*J*_) branches, causing quenching of the MMCT state. The latter parameter is the dominant one for the different behavior of CaS:Eu,Tb and SrS:Eu,Tb.

The above analysis can be expanded to devise guidelines for finding other phosphors that exhibit MMCT luminescence. To achieve this, the ground state potential energy surface should cross the emitting MMCT level at a sufficiently large *Q*_et_ value. For this, small curvatures are required, which translates into small vibrational frequencies, a property that is not only typical for sulfide hosts^30,70^, but also for selenides^30^, nitrides^71^, chlorides^72^, bromides^72^, and iodides^73^. Additionally, the crossing between the ground state and the MMCT level can be shifted away from the MMCT minimum by decreasing the horizontal offset between both parabolas, that is, by decreasing the equilibrium *Q*_et_ value. This value is proportional to the change in lanthanide–ligand bond length upon CT and to the square of the coordination number of the lanthanide^67^. MMCT luminescence will hence be more probable in hosts with a small site for the lanthanide as these experience smaller bond length changes. Ca-based hosts, preferably with a low coordination number, are hence more desirable with respect to Sr- or Ba-based hosts.

When the chemical composition of host compounds is engineered to accomplish MMCT luminescence, also the vertical offset, and hence the MMCT emission energy, is expected to be affected. Indeed, the vertical offset is given by the difference of the ionization potential (IP) of the donor and the electron affinity (EA) of the acceptor, supplemented with the Coulomb and exchange interaction between both ions^67^, and these parameters are host dependent. A more radical manipulation of the vertical offset can be achieved by substituting the lanthanide ions. A rough idea on the effect of host modification and lanthanide substitution on the vertical offset can be acquired by consultation of the lengthy empirical data on IP’s and EA’s of lanthanide ions and their systematic behavior^74,75^.

In summary, the presence or absence of MMCT luminescence, and the emission energy is affected by three parameters: the local vibrational frequency, bond length change, and the selected lanthanide pair. A perfect balance between these parameters is required to achieve MMCT luminescence as in the case of CaS:Eu^2+^,Tb^3+^.

In this combined experimental–theoretical study, broadband IR emission in CaS:Eu^2+^,Tb^3+^ is reported, characterized, and explained. The emission spectrum overlaps with the regular red Eu^2+^5*d*−4*f* luminescence and ranges up to 1200 nm. Importantly, it can be efficiently pumped with long-wavelength visible light.

Concentration-dependent and microscopy experiments showed that the IR emission is caused by a cooperative effect between Eu and Tb. Ab initio multiconfigurational calculations support that the IR-emitting state is a Eu^3+^–Tb^2+^ MMCT state whose local structure differs significantly from the Eu^2+^–Tb^3+^ ground state.

The type of host has a critical influence on the properties of the MMCT luminescence, as shown by the fact that the IR emission is quenched in the similar compound SrS. This behavior was explained by the ab initio calculations, which show that the location of MMCT states, and hence their luminescence properties can be fine-tuned by tweaking a few parameters. These are experimentally accessible by altering the anions and cations in the host, or the lanthanide pair, namely the local vibrational frequencies and structural rearrangement upon MMCT, as well as the IP and electron affinities of the dopants.

The CaS:Eu,Tb phosphor was used to construct a broadband IR pc-LED for spectroscopic applications in smart electronics, food safety, and medicine. The IR emission of the LED covers a 430-nm-wide spectral range in the red and near-IR. Moreover, this is achieved with an IR output radiant flux of 38 mW, surpassing current state of the art.

## Methods

### Experimental method

Powders of CaS and SrS, doped with Eu and/or Tb were prepared by a solid-state synthesis, using high-purity CaS (Alfa Aesar, 99.9%), SrS (Alfa Aesar, 99.9%), EuF_3_ (Alfa Aesar, 99.95%), and TbF_3_ (Alfa Aesar, 99.9%) as precursors. Stoichiometric quantities were weighed, mixed, and subsequently heat treated in a tube furnace for 2 h at 1000 °C under a constant flow of H_2_S gas. After the heat treatment, the samples were allowed to cool naturally. Finally, the samples were slightly ground and stored in an inert atmosphere. All samples were phase pure, as verified by powder XRD (see Supplementary Fig. 1). Doping concentrations that were reported are molar concentrations with respect to the cation, for example, CaS:Eu_0.010_Tb_0.001_ is used for Ca_0.989_Eu_0.010_Tb_0.001_S.

PL emission and excitation spectra were measured on an Edinburgh FS920, using a 450 W xenon arc lamp as excitation source and equipped with a Hamamatsu R928P red-sensitive photomultiplier (wavelength range from 200 to 850 nm) and a Ge IR detector (700–1600 nm). Temperature-dependent PL was measured with the same spectrometer, equipped with a cryostat (Oxford Instruments Optistat CF).

The microscopy results were obtained with a Hitachi S-3400N SEM. A Thermo Scientific Noran System 7 EDX was used for chemical analysis and an optical fiber to collect the CL, which was subsequently analyzed by an Acton SP2300 monochromator and detected by a ProEM 1600 EMCCD (both Princeton Instruments). All shown spectra were properly calibrated for the spectral sensitivity of the various detectors.

An IR pc-LED was constructed using a blue pumping LED. For this a Xicato XTM LED module was used, operated at a constant current of 130 mA, corresponding to a voltage of 16.7 V. Its spectral radiant flux was obtained using a Thorlabs S401C thermal power meter.

### Computational method

Diabatic potential energy surfaces and derived configurational coordinate diagrams were calculated for metal-to-metal electron transfer states of Eu^2+^/Tb^3+^ mixed valence pairs in CaS and SrS, using the results of independent embedded cluster calculations as proposed in refs. ^22,23^.

The electronic structures of the electron donor (Eu) and acceptor (Tb) octahedral embedded clusters (LnS_6_M_6_)^2+^ and (LnS_6_M_6_)^3+^ (M = Ca, Sr) were obtained with the suite of programs MOLCAS,^76^ using *D*_2*h*_ symmetry, in two-step spin–orbit coupling state-average restricted-active-space self-consistent-field (SA-RASSCF)/multi-state second-order perturbation theory (MS-RASPT2)/restricted-active-space state-interaction spin–orbit (RASSI-SO) DKH calculations. In a first step, the spin–orbit-free many-electron relativistic second-order DKH Hamiltonian^77,78^ was used to perform all-electron calculations using the same type of basis sets as in ref. ^31^: Gaussian atomic natural orbital relativistic basis sets ANO-RCC for S^79^, Eu, and Tb^80^, with respective contractions (17*s*12*p*5*d*)/[6*s*5*p*3*d*] (quadruple-zeta with polarization without *f*-functions quality) and (25*s*22*p*15*d*11*f*4*g*2*h*)/[9*s*8*p*5*d*4*f*3*g*2*h*] (quadruple-zeta with polarization quality). In addition, the six-electron valence was explicitly added to the six alkaline earth metal ions next to the sulfur ligands in the [100] directions using adapted ANO-RCC basis sets, Ca(20*s*16*p*6*d*)/[3*s*4*p*1*d*] and Sr(23*s*19*p*12*d*)/[3*s*4*p*1*d*]. The inner shells of these ions were frozen, using a [Mg] and [Zn] core for Ca and Sr, respectively.

First, SA-RASSCF^81–83^ calculations were performed, allowing all possible occupations in the Ln 4*f* shells and up to four electrons in the Ln 5*d*, 6*s*, and 5*f* shells, in order to account for the so-called double-shell effect^84^. Following states were obtained: those of the 4*f*^6^ and 4*f*^8^ configurations of Eu^3+^ and Tb^3+^ for which 2*S* + 1 = 7, 5, those of the 4*f*^7^, 4*f*^6^5*d*^1^, and 4*f*^8^5*d*^1^ of Eu^2+^ and Tb^2+^ for which 2*S* + 1 = 8, 6 and those of the Tb^2+^ 4*f*^9^ configuration for which 2*S* + 1 = 6. States with lower multiplicities were not considered because they are not expected to influence the lowest part of the energy spectrum that is of interest. Subsequently, MS-RASPT2^85–88^ calculations allowed to correlate all cluster valence electrons, except the 4*d* electrons of the lanthanides. A standard IPEA value (0.25 a.u.)^89^ and an imaginary shift of 0.15 a.u. (Eu) or 0.50 a.u. (Tb) was used. Second, the AMFI approximation of the DKH spin–orbit coupling operator was added to the Hamiltonian^90^ and RASSI-SO^91,92^ calculations were performed. Here, all states of a given cluster computed in the first step were allowed to interact.

In all calculations, the clusters were embedded in ab initio model potentials (AIMPs)^93^ that include Coulomb, exchange, and Pauli repulsion interactions from the CaS and SrS host lattices obtained in ref. ^31^ from self-consistent embedded ions^94^ Hartree–Fock calculations^76^. Figure 5 shows how the small cluster is embedded in AIMPs and point charges.

## Supplementary information

Supplementary Information

## Data Availability

The data sets generated during and/or analyzed during the current study are available from the corresponding author on reasonable request.

## Code availability

Multiconfigurational ab initio calculations were performed using MOLCAS.
