# Supplementary material for: Broadband infrared LEDs based on europium-to-terbium charge transfer luminescence
Source: Nat Commun. 2020 Jul 20;11:3647. doi: 10.1038/s41467-020-17469-x (PMC7371692; doi:10.1038/s41467-020-17469-x)
Supplement: Supplementary file 1 — Supplementary Information [file 41467_2020_17469_MOESM1_ESM.pdf]

Broadband infrared LEDs based on europium-to-terbium charge transfer luminescence

Supplementary Information

Joos *et al.*

Broadband infrared LEDs based on europium-to-terbium charge transfer luminescence
Supplementary Information

Jonas J. Joos,[1,2,*] David Van der Heggen,[1,2] Lisa I. D. J. Martin,[1,2] Lucia Amidani,[3,†] Philippe F. Smet,[4,5] Zoila Barandiarán,[6,7,8] and Luis Seijo[6,7,8]

[1] *LumiLab, Department of Solid State Sciences, Ghent University, 9000 Gent, Belgium*
[2] *Center for Nano- and Biophotonics (NB Photonics), Ghent University, 9000 Gent, Belgium*
[3] *European Synchrotron Radiation Facility (ESRF), 38000 Grenoble, France*
[4] *LumiLab, Department of Solid State Sciences, Ghent University, Gent, Belgium*
[5] *Center for Nano- and Biophotonics (NB Photonics), Ghent University, Gent, Belgium*
[6] *Departamento de Química, Universidad Autónoma de Madrid, 28049 Madrid, Spain*
[7] *Instituto Universitario de Ciencia de Materiales Nicolás Cabrera, Universidad Autónoma de Madrid, 28049 Madrid, Spain*
[8] *Condensed Matter Physics Center (IFIMAC), Universidad Autónoma de Madrid, 28049 Madrid, Spain*

(Dated: June 19, 2020)

SUPPLEMENTARY DISCUSSION

X-ray diffraction

Supplementary Figure 1 shows the powder X-ray diffraction (XRD) patterns, measured for the various CaS:Eu,Tb phosphors that were prepared. These were obtained with a Siemens D5000 diffractometer (40 kV, 40 mA) using Cu$K\alpha$ radiation. No impurity phases can be identified from these measurements.

X-ray spectroscopy

X-ray spectroscopy was performed at the ID26 beamline at the European Synchrotron Radiation Facility (ESRF)[1]. High Energy Resolution Fluorescence Detected X-ray Absorption Near Edge Structure (HERFD-XANES) spectra were recorded on the Eu L_3 edge (6.9769 keV) and on the Tb L_3 edge (7.5140 keV). Compared to regular XANES, with HERFD-XANES only a small bandwidth near the maximum of a characteristic X-ray emission line is integrated, yielding an appreciable improvement in energy resolution[2]. For this, an X-ray emission spectrometer based on Rowland geometry was used. The spectrometer employs an array of four spherically-bent crystal analyzers to select by means of the Bragg's law only the X-rays of the desired energy. Ge(333) crystals were aligned to the Eu L_{α_1} line (5.846 keV), or Ge(330) crystals to the Tb $L\alpha_1$ line (6.2728 keV). The incident energy was selected with a Si(311) double crystal monochromator. Pressed pellets of CaS:Eu,Tb, diluted in boron nitride (BN) in a 1:10 mass ratio were used as sample. X-ray induced valence changes or degradation was excluded during the measurement.

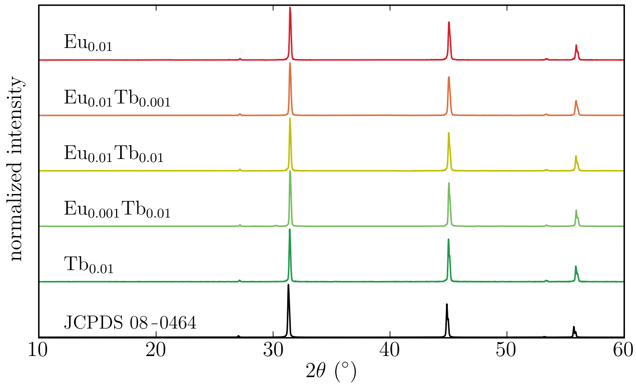

Supplementary Figure 1. XRD measurements of the prepared CaS:Eu,Tb powders, compared with the reference pattern JCPDS 08-0464 for CaS.

Supplementary Figure 2 shows the resulting HERFD-XANES spectra for the CaS:$Eu_{0.03}Tb_{0.03}$ sample, compared to the reference compounds EuS (EuII), EuF_3 (EuIII), TbF_3 (TbIII) and Tb_4O_7 (TbIII and TbIV). From these measurements, it is clear that

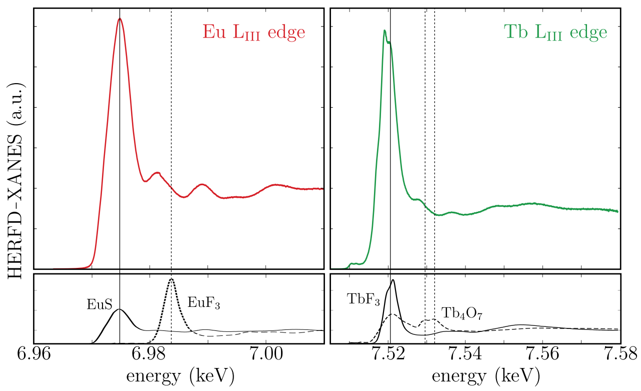

Supplementary Figure 2. Eu (left) and Tb (right) L_3 HERFD-XANES spectra of CaS:$Eu_{0.03}Tb_{0.03}$, compared to the reference compounds EuS, EuF_3, TbF_3 and Tb_4O_7.

the no detectable amount of Eu^{3+} is present in the $CaS{:}Eu_{0.03}Tb_{0.03}$ sample. The same is true for Tb^{4+}. This indicates that, within the experimental detection limits, the prepared CaS:Eu,Tb powders contain solely Eu^{2+} and Tb^{3+}.

Dopant distribution

Scanning electron microscopy (SEM), complemented with Energy-dispersive X-ray Spectroscopy is applied to verify the dopant distribution inside the prepared phosphors. For this, a Hitachi S-3400N scanning electron microscope, equipped with a Thermo Scientific Noran System 7 energy-dispersive X-ray detector is used.

Supplementary Figure 3 shows a SEM-EDX scan on a 125 × 90 μm^2 area of the CaS:1%Eu,1%Tb powder. EDX maps for Eu and Tb indicate that the dopants are well distributed in the powder, apart from some small µm sized grains with higher Eu or Tb concentrations that can be found here and there. Both dopants mix appreciably as shown in Supplementary Figure 3(d) where simultaneous detection leads to a yellow color in the map. Finally, the dopant concentration was locally quantified by Phi-Rho-Z ($\phi(\rho z)$) quantification[3], and collected in histograms (see Supplementary Figure 3(e))[4]. It is found that the doping concentrations amount to 0.014 ± 0.008 and 0.014 ± 0.009 for Eu and Tb, respectively. The relatively narrow distributions, quantified by the above-mentioned standard deviations, demonstrate the overall homogeneity of the doping within experimentally achievable limits[4–6].

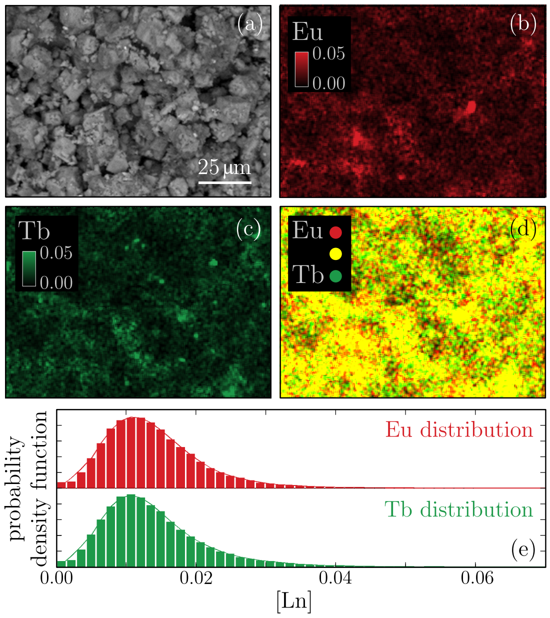

Supplementary Figure 3. Backscattered electron image (a), and EDX maps for Eu (b) and Tb (c) for CaS:1%Eu,1%Tb. The map in (d) displays the mixing of Eu and Tb and the histograms (e) quantify the local concentrations of both dopants.

IR emission vs. precursors / charge compensation

To exclude that the observed broadband IR emission is a spurious effect, induced by the used precursors, several different syntheses were performed using different precursors for the lanthanides. Supplementary Figure 4 shows the emission spectra of different CaS:1%Eu,1%Tb powders. This illustrates that the IR emission is indeed always present, safely excluding that it is induced as a consequence of the used precursors.

Although the presence of the IR emission is not affected by the used precursor, small variations in the ratio of the red and IR emission intensities can however be seen, indicating that the precursors have some effect on the distributions and incorporation of the dopants, possibly due to different charge compensating mechanisms, the flux effect of some precursors, notably fluorides[7–10], or other hidden variables that are only of secondary importance.

To acquire more information about the role of charge compensators, especially for the trivalent Tb^{3+} ion, sodium (Na^+) was selected as codopant. It was found that the addition of Na^+ strongly increases the IR:red ratio (see Supplementary Figure 4), however at the cost of a strongly decreased overall quantum efficiency (see further). This could be expected as it is known that Na^+ will stabilize Eu^{3+} as well[11], which is undesired because Eu^{2+}-Eu^{3+} pairs will be generated in addition to Eu^{2+}-Tb^{3+} pairs. The former pairs will negatively impact the quantum efficiency because of intervalence charge transfer (IVCT) quenching[12,13]. As a further consequence of the Na^+ addition, fewer isolated Eu^{2+}centers will exist, explaining the reduced red component in the spectrum. It is hence concluded that the addition of Na^+ charge compensators negatively affects the luminescent properties of the Eu,Tb co-doped phosphor because it stabilizes Eu^{3+}. It has however no direct impact on the emission

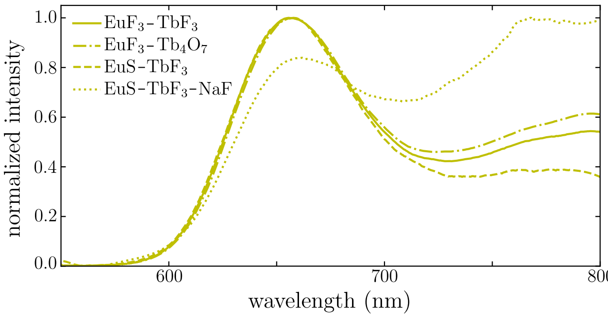

Supplementary Figure 4. Photoluminescence emission spectra of Eu^{2+} and Tb^{3+} codoped CaS phosphors, prepared with a solid state reaction, using different precursors for Eu and Tb.

spectrum of the IR-emitting Eu^{2+}-Tb^{3+}centers. As the broadband IR luminescence is the main topic of this article, the role of precursors or intrinisic or extrinsic charge compensation will not be elaborated further.

Additional Photoluminescence spectra

High doping concentration

Supplementary Figure 5 displays the emission spectra for CaS powders with a fixed Eu concentration of 3% and an increasing Tb concentration. It is found that the IR:red ratio is maximal for CaS:3%Eu,3%Tb and decreases again for higher Tb concentrations. This result confirms the SEM-CL mapping (Figure 4) where it was found that the IR:red ratio is limited to the one for CaS:3%Eu,3%Tb. For higher doping concentrations, it is likely that defect clusters are formed containing multiple lanthanide dopants in a small region of the crystal, probably in addition to other defects. These complex structures strongly affect the local crystal structure, as well as the associated excited state landscape. It is hence not surprising that the radiative MMCT decay channel gets quenched for these high concentrations.

It should also be noted that the red emission is always found in addition to the IR emission. This equilibrium between red and IR emissions implies that the IR emission is presumably not (only) the result of an energy transfer from Eu^{2+} to another emitting center because it would then be expected that the Eu^{2+} emission would vanish completely if the concentrations are stretched sufficiently[14,15].

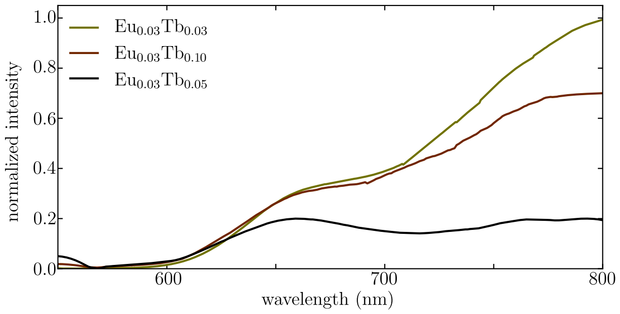

Supplementary Figure 5. Photoluminescence emission spectra of Eu^{2+} and Tb^{3+} codoped CaS (top) and SrS (bottom) phosphors. The spectra were collected upon 285 nm excitation. All spectra were measured at room temperature.

Low temperature

Supplementary Figure 6 displays the photoluminescence spectra of CaS:1%Eu,1%Tb measured at 10 K. Overall, the same spectral features and assignments apply as at room temperature (RT, see main text). In contrast to the RT spectra, here some phonon fine structure can be resolved. The zero-phonon line is indicated by the black arrow.

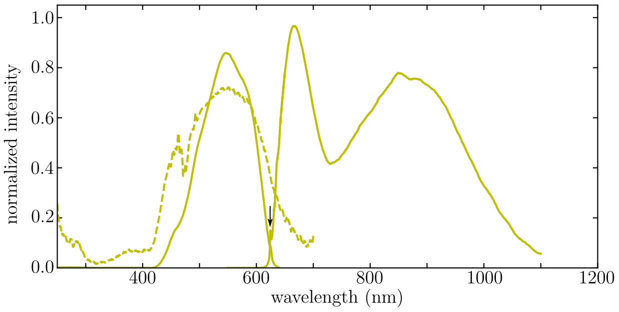

Supplementary Figure 6. Photoluminescence emission and excitation spectra of CaS:1%Eu,1%Tb, measured at 10 K. The emission spectrum was measured upon 490 nm excitation, the excitation spectra were measured for 670 nm (solid line) and 850 nm (dashed line) emission.

Diffuse reflectance spectra

The diffuse reflectance spectra of several CaS:Eu,Tb powders, shown in Supplementary Figure 7, were obtained with a Perkin Elmer Lambda 1050 UV-Vis-NIR spectrophotometer, equipped with an integrating sphere. The sample was kept in a powder sample holder and covered by a quartz window during the measurement.

The absorption band in the range 650 nm to 450 nm is due to the numerous Eu^{2+} $4f^7 \rightarrow 4f^6 5dt_{2g}$ transitions. Below 400 nm, fundamental absorption of the CaS host is visible, overlapping with the broad Eu-to-Tb MMCT absorption band. For more information on spectral assignments, see main text and Ref. 16.

As evidenced from the diffuse reflectance spectra, the prepared powders suffer from significant greying, showing reflectance values of 35-40% in the long wavelength region where no Eu^{2+} related absorptions are expected. Furthermore, the Eu^{2+} $4f^7 - 4f^6 5dt^1_{2g}$ absorption strength in the 500 nm region does not straightforwardly scale with the Eu^{2+} concentration. In part, this can be explained by the different greying for the different powders. Furthermore, the crystallization, dopant incorporation and hence absorption strength of inorganic phosphors, notably sulfides[7,8], is strongly affected by fluxing effects during the solid state synthesis, caused by, among others, fluoride precursors for the lanthanide dopants[9,10]. These observations indicate that there is margin for improving the efficiency of the prepared sulfide phosphors, possibly by carefully selecting fluxing agents.

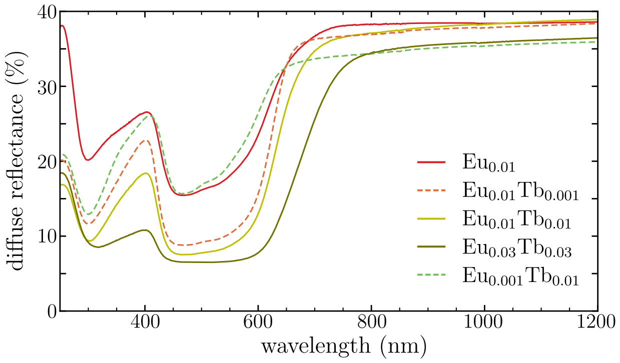

Supplementary Figure 7. Diffuse reflectance spectra of Eu^{2+} and Tb^{3+} codoped CaS phosphors.

Photoluminescence quantum efficiency

Photoluminescence quantum efficiencies (QE) were measured inside an integrating sphere (152 mm, spectralon coated), using an LED ($\lambda_{max} = 470$ nm) for excitation. The reflection of the LED and the red and IR luminescence were collected by an optical fiber, analyzed by an Acton SP2300 monochromator and recorded by a ProEM 1600 EMCCD camera (both Princeton Instruments). The detection setup was properly calibrated for its spectral response[17].

Supplementary Figure 8 shows the resulting internal QE's, *i.e.* the ratio of the number of emitted (red+IR) photons to the number of absorbed (blue) photons as a function of the Eu concentration:

$$QE_{int} = \frac{N_{em}}{N_{abs}} = \frac{N_{em}}{N_{inc} - N_{refl}}. \quad (1)$$

It is clear that these phosphors are prone to concentration quenching, showing a drop in efficiencies for [Eu] $>$ 1%. This is similar to other Eu-doped phosphors[8]. Furthermore, this figure suggests that also the terbium content, [Tb], affects the efficiencies: upon increasing the terbium content in CaS:1%Eu,Tb, QE_{int} goes from 35% (no Tb) to 25% (0.1% Tb) to 10% (1% Tb). These results confirm the *ab initio* calculations that predict an additional non-radiative decay channel, *i.e.* outside cross-over of the MMCT states with the ground state of the Eu^{2+}-Tb^{3+} pair, in addition to the IR MMCT emission. A similar trend, however of a smaller magnitude, is found for SrS:Eu,Tb where the same outside cross-over fully quenches the MMCT state (see main text for details).

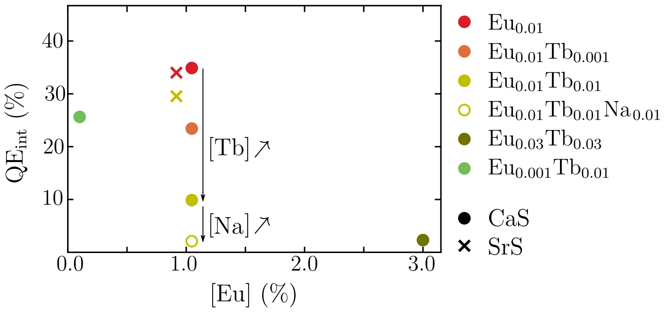

Supplementary Figure 8. Internal quantum efficiencies (QE_{int}) of the different Eu^{2+} and Tb^{3+} codoped CaS (circles) and SrS (crosses) phosphors.

Luminescence decay

Luminescence decay profiles were measured using a pulsed Optical Parametric Oscillator (OPO) laser (Ekspla NT342B), set at 470 nm, as excitation source in combination with an Andor intensified CCD. The resulting profiles for the red and IR emission of $CaS{:}Eu_{0.01}Tb_{0.01}$ are shown in Supplementary Figure 9. These profiles were fit using the sum of three exponentials,

$$I(t) = I_1 e^{-t/\tau_1} + I_2 e^{-t/\tau_2} + I_3 e^{-t/\tau_3}. \quad (2)$$

The obtained parameters are given in Supplementary Table 1. The short decay component, τ_1, of the red Eu^{2+} $4f^6 5d^1 \rightarrow 4f^7$ and IR MMCT emissions are of the same order of magnitude, around 500 ns. This corresponds to the expected radiative decay rate of Eu^{2+} in CaS, which is relatively fast for Eu^{2+} due to the high refractive index of CaS[18].

Furthermore, similar for both emission bands, τ_2 and τ_3 correspond to delayed luminescence with characteristic times of the order of 4 and 35 µs, respectively. This indicates that presumably some trapping and detrapping is involved. This observation is in correspondence with the TL peak that shows up around and above room temperature in the thermal quenching measurement (see Figure 5). The trapping/detrapping kinetics are not further investigated here as they fall out of the scope of the MMCT luminescence that we report here.

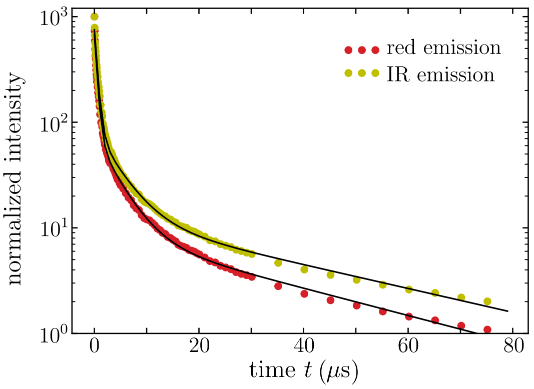

Supplementary Figure 9. Luminescence decay profiles of the red and IR emission bands of $CaS{:}Eu_{0.01}Tb_{0.01}$ and the corresponding fits (in black).

Supplementary Table 1. Fit parameters of the photoluminescence decay profiles of $CaS{:}Eu_{0.01}Tb_{0.01}$ according to Supplementary Equation 2. Times in μs.

	I_1	τ_1	I_2	τ_2	I_3	τ_3	R^2
red	0.600	0.487	0.068	4.035	0.009	33.60	0.998
IR	0.645	0.537	0.075	4.430	0.013	38.49	0.990

Electronic structure of MS:Tb^{3+} and MS:Tb^{2+} (M=Ca,Sr)

In order to construct the diabatic configurational coordinate diagram (Figure 5), the configurational coordinate diagrams along the fully symmetric breathing mode for the isolated Eu^{2+}, Eu^{3+}, Tb^{3+} and Tb^{2+} defects are needed. This is calculated with the *ab initio* multiconfigurational SA-RASSCF/MS-RASPT2/RASSI-SO approach, as described in the Methods section and in more detail in Ref.[16]. The detailed analysis of the electronic structure of Eu^{2+} and Eu^{3+} doped CaS and SrS can be found in Ref.[16] and its supplementary info. An analogous analysis for Tb^{3+} and Tb^{2+} doped CaS and SrS can be found here.

The results of the spin-free (SA-RASSCF/MS-RASPT2) calculations can be found in Supplementary Tables 2-3 and in Supplementary Figures 10-13. The results after spin-orbit coupling was included at RASSI-SO level and can be found in Supplementary Tables 6-7 and in Supplementary Figures 14-15.

The excited state landscape of the Tb^{3+} doped sulfides corresponds to the well-known structure; the ^7F_J ($J = 6-0$) multiplet covers the lowest 6000 cm^{-1} after which a gap is found up to the $^5D_4$ levels around 20000 cm^{-1}. Another small gap of 5000 cm^{-1} is then found before the $^5D_3$ level, the second emitting level of isolated Tb^{3+} ions. Above this level, a very dense set of spin-quintets is found. In our calculations, the levels originating from the ^5L and ^5G terms are calculated in addition to the ^7F and ^5D terms.

In contrast to Tb^{3+}, divalent terbium, Tb^{2+}, is a more exotic ion, which is likely not stable in CaS nor SrS. However, in order to construct the MMCT excited states of a Eu^{2+}-Tb^{3+} pair, the computation of the ground and excited states of Tb^{2+} in CaS and SrS is needed. Our results show that the $4f^8 5d^1$ configuration forms the ground state for the Tb^{2+} ion in CaS. A similar picture is found for SrS at SA-RASSCF level (see Supplementary Fig. 11), while the $4f^9$ states undergo a significant shift with respect to the $4f^8 5d^1$ states upon including the non-static correlation (MS-RASPT2, see Supplementary Figure 13), leading to a near-degeneracy for the lowest $4f^9$ and the lowest $4f^8 5d^1$ levels. Even though it is expected that the $4f^8 5d^1 - 4f^9$ energy difference decreases when going from CaS to SrS from extrapolating spectroscopic information from Eu^{2+} to other divalent lanthanides such as Tb^{2+} based on empirical models[19,20], the large decrease of the $4f^8 5d^1 - 4f^9$ gap at MS-RASPT2 level is surprising. In any case, the value of the $4f^8 5d^1 - 4f^9$ gap does not affect any conclusions concerning the MMCT emission reported here, as this is determined predominantly by the Tb^{3+} electron affinity, breathing mode vibrational frequencies and lanthanide-ligand bond lengths (see main text).

The $4f^8 5d^1$ manifold is a very dense set of energy levels and no energy gaps worth mentioning can be found in the explored energy range. For this reason, Tb^{2+}, if it

would exist as isolated impurity in CaS or SrS, is not expected to be luminescent. Furthermore, a rather weak absorption strength is expected because no spin-allowed interconfigurational $4f^85d^1 \rightarrow 4f^9$ transitions from the ground state spin-octet exist as the highest multiplicity of the $4f^9$ manifold is 6. Regardless of the rarity of Tb^{2+} in literature, some claims of stable Tb^{2+} impurities in crystalline hosts can be found in literature, typically after a sample was exposed to heavy gamma irradiation[21,22].

* jonas.joos@UGent.be
† Current affiliation: Helmholtz-Zentrum Dresden-Rossendorf, 01314 Dresden, Germany

[1] C. Gauthier, V. A. Sole, R. Signorato, J. Goulon, and E. Moguiline, "The ESRF Beamline ID26: X-ray Absorption on Ultra Dilute Sample," J. Synchrotron Radiat. **6**, 164–166 (1999).

[2] P. Glatzel, T. C. Weng, K. Kvashnina, J. Swarbrick, M. Sikora, E. Gallo, N. Smolentsev, and R. A. Mori, "Reflections on Hard X-Ray Photon-In/Photon-Out Spectroscopy for Electronic Structure Studies," J. Electron Spectrosc. **188**, 17–25 (2013).

[3] R. H. Packwood and J. D. Brown, "A Gaussian Expression to Describe $\phi(\rho z)$ Curves for Quantitative Electron Probe Microanalysis," X-Ray Spectrom. **10**, 138–146 (1981).

[4] L. I. D. J. Martin, D. Poelman, P. F. Smet, and J. J. Joos, "Microscopic Study of Dopant Distribution in Europium Doped $SrGa_2S_4$: Impact on Thermal Quenching and Phosphor Performance," ECS J. Solid State Sci. Technol. **7**, R3052–R3056 (2018).

[5] L. L. Noto, S. K. K. Shaat, D. Poelman, P. F. Smet, L. Martin, M. Y. A. Yagoub, S. M. Dhlamini, O. M. Ntwaeaborwa, and H. C. Swart, "Photoluminescence and Phase Related Cathodoluminescence Dynamics of Pr^{3+} Doped in a Double Phase of $ZnTa_2O_6$ and $ZnAl_2O_4$," Ceramics International **42**, 5497–5503 (2016).

[6] W. Y. Li, P. F. Smet, L. I. D. J. Martin, C. Pritzel, and J. S. auf der Günne, "Doping Homogeneity in Co-Doped Materials Investigated at Different Length Scales," Phys. Chem. Chem. Phys. **22**, 818–825 (2020).

[7] K. Kato and F. Okamoto, "Preparation and Cathodoluminescence of CaS:Eu and $Ca_{1-x}Sr_xS$:Eu Phosphors," Jpn. J. Appl. Phys. **22**, 76–78 (1983).

[8] J. J. Joos, K. W. Meert, A. B. Parmentier, D. Poelman, and P. F. Smet, "Thermal Quenching and Luminescence Lifetime of Saturated Green $Sr_{1-x}Eu_xGa_2S_4$ Phosphors," Opt. Mater. **34**, 1902–1907 (2012).

[9] L. Zhang, Z. Lu, P. D. Han, J. J. Lu, N. C. Xu, L. X. Wang, and Q. T. Zhang, "The Evolution and Role of NH_4Cl Flux Used to Synthesize Sr_2SiO_4:Dy^{3+} Phosphor by Solid-State Reaction Method," J. Am. Ceram. Soc. **95**, 3871–3877 (2012).

[10] L. H. Liu, R.-J. Xie, C. N. Zhang, and N. Hirosaki, "Role of Fluxes in Optimizing the Optical Properties of $Sr_{0.95}Si_2O_2N_2$:$0.05Eu^{2+}$ Green-Emitting Phosphor," Materials **6**, 2862–2872 (2013).

[11] N Yamashita, S. Fukumoto, S. Ibuki, and H. Ohnishi, "Photoluminescence of Eu^{2+} and Eu^{3+} Centers in CaS:Eu,Na Phosphors," Jpn. J. Appl. Phys. **32**, 3135–3139 (1993).

[12] J. J. Joos, L. Seijo, and Z. Barandiarán, "Direct Evidence of Intervalence Charge-Transfer States of Eu-Doped Luminescent Materials," J. Phys. Chem. Lett. **10**, 1581–1586 (2019).

[13] I. Neefjes, J. J. Joos, Z. Barandiarán, and L. Seijo, "Mixed-Valence Lanthanide-Activated Phosphors: Invariance of the Intervalence Charge Transfer (IVCT) Absorption Onset across the Series," J. Phys. Chem. C **124**, 2619–2626 (2020).

[14] D. L. Dexter, "A Theory of Sensitized Luminescence in Solids," J. Chem. Phys. **21**, 836–850 (1953).

[15] M. Bettinelli, A. Speghini, F. Piccinelli, J. Ueda, and S. Tanabe, "Energy Transfer Processes in $Sr_3Tb_{0.90}Eu_{0.10}(PO_4)_3$," Opt. Mater. **33**, 119–122 (2010).

[16] J. J. Joos, P. F. Smet, L. Seijo, and Z. Barandiarán, "Insights into the Complexity of the Excited States of Eu-Doped Luminescent Materials," Inorg. Chem. Front. **7**, 871–888 (2020).

[17] S. Leyre, E. Coutino-González, J. J. Joos, J. Ryckaert, Y. Meuret, D. Poelman, P. F. Smet, G. Durinck, J. Hofkens, G. Deconinck, and P. Hanselaer, "Absolute Determination of Photoluminescence Quantum Efficiency Using an Integrating Sphere Setup," Rev. Sci. Instrum. **85**, 123115 (2014).

[18] S. H. M. Poort, A. Meijerink, and G. Blasse, "Lifetime measurements in Eu^{2+}-doped host lattices," J. Phys. Chem. Solids **58**, 1451–1456 (1997).

[19] A. B. Parmentier, J. J. Joos, P. F. Smet, and D. Poelman, "Luminescence of Ytterbium in CaS and SrS," J. Lumin. **154**, 445–451 (2014).

[20] D. C. Rodriguez Burbano, S. K. Sharma, P. Dorenbos, B. Viana, and J. A. Capobianco, "Persistent and Photostimulated Red Emission in CaS:Eu^{2+},Dy^{3+} Nanophosphors," Adv. Opt. Mater. **3**, 551–557 (2015).

[21] D. S. McClure and Z. Kiss, "Survery of Spectra of Divalent Rare-Earth Ions in Cubic Crystals," J. Chem. Phys. **39**, 3251–3257 (1963).

[22] J. L. Merz and P. S. Pershan, "Charge Conversion of Irradiated Rare-Earth Ions in Calcium Fluoride. I," Phys. Rev. **162**, 217–235 (1967).

Supplementary Table 2. Spectroscopic constants of the ground and excited states of Tb^{3+} and Tb^{2+} -doped CaS octahedral defects calculated using the relativistic spin-free second order Douglas-Kroll-Hess Hamiltonian with AIMP CaS embedding. The embedded cluster RASSCF results include basic bonding interactions and $4f$–shell radial correlation; the MS-RASPT2 ones include also dynamic correlation of 99 or 98 valence electrons in the Tb $5s, 5p$, S $3s, 3p$, Ca $3p$ closed shells and Tb $4f, 5d, 6s$ open shells. Tb–S bond distances ($d_{\mathrm{Tb-S},e}$ in Å), TbS_6 breathing mode harmonic vibrational frequencies ($\omega_{a_{1g}}$ in cm^{-1}), and minimum-to-minimum energy differences (T_e in cm^{-1}) are given. See Figs. 12, 13 and text for details.

	RASSCF			MS-RASPT2		
term	$d_{\mathrm{Tb-S},e}$	$\omega_{a_{1g}}$	T_e	$d_{\mathrm{Tb-S},e}$	$\omega_{a_{1g}}$	T_e
			Tb^{3+}-doped CaS			
$4f^8(^7F)$ [a]						
$1\ ^7A_{2g}$	2.744	317	0	2.707	306	0
$1\ ^7T_{2g}$	2.744	317	720	2.708	306	579
$1\ ^7T_{1g}$	2.745	317	728	2.708	306	977
$4f^8(^5D)$						
$1\ ^5T_{2g}$	2.744	317	25447	2.704	304	21556
$1\ ^5E_g$	2.744	317	25441	2.704	304	21595
$4f^8(^5L,^5G)$						
$2\ ^5T_{2g}$	2.744	317	29627	2.704	303	26060
$2\ ^5E_g$	2.744	317	29466	2.704	306	26297
$1\ ^5T_{1g}$	2.744	317	29432	2.704	303	26304
$1\ ^5A_{1g}$	2.744	317	29425	2.704	304	26397
$2\ ^5T_{1g}$	2.744	317	29678	2.704	303	26675
$3\ ^5E_g$	2.744	317	29730	2.704	307	26835
$2\ ^5A_{1g}$	2.744	317	30307	2.703	302	26837
$3\ ^5T_{2g}$	2.744	317	29740	2.704	304	27127
$3\ ^5T_{1g}$	2.744	317	30341	2.704	304	27298
$4\ ^5E_g$	2.744	317	30291	2.704	312	27389
$4\ ^5T_{2g}$	2.744	317	30210	2.704	304	27431
			Tb^{2+}-doped CaS			
			$4f^8(5d+6s)^1$			
$4f^8(^7F)5dt_{2g}^1$						
Octets						
$1\ ^8A_{1g}$	2.878	296	0	2.834	277	0
$1\ ^8T_{1g}$	2.878	297	937	2.833	277	532
$1\ ^8E_g$	2.878	296	1262	2.833	277	1046
$1\ ^8T_{2g}$	2.877	296	1900	2.832	277	1596
$1\ ^8A_{2g}$	2.877	296	2185	2.832	277	2324
$2\ ^8T_{2g}$	2.876	296	4259	2.831	277	3221
$2\ ^8T_{1g}$	2.876	296	4252	2.829	277	3324
$2\ ^8E_g$	2.875	296	4353	2.831	278	3363
$3\ ^8T_{1g}$	2.873	295	7201	2.827	276	5833
Sextets						
$1\ ^6A_{2g}$	2.872	295	9306	2.829	276	8260
$1\ ^6T_{2g}$	2.872	295	9630	2.827	277	8538
$1\ ^6T_{1g}$	2.870	295	9849	2.826	276	8630
$1\ ^6A_{1g}$	2.872	295	10819	2.827	276	8751
$1\ ^6E_g$	2.871	295	10790	2.827	276	9338

2 $^6T_{1g}$	2.872	295	10835	2.827	276	9426
2 $^6E_{g}$	2.870	295	12600	2.825	276	10416
2 $^6T_{2g}$	2.869	295	12406	2.825	277	10693
3 $^6T_{1g}$	2.869	294	13451	2.825	276	11344
$4f^8(^7F)(5de_g^1 + 6s_{a_{1g}}^1)$						
Octets						
3 $^8T_{2g}$	2.912	297	19170	2.869	270	19672
3 $^8E_{g}$	2.910	295	21656	2.867	274	21916
4 $^8T_{1g}$	2.910	295	21921	2.867	269	22245
4 $^8T_{2g}$	2.910	294	23255	2.867	268	22808
5 $^8T_{1g}$	2.907	293	26530	2.862	265	25860
2 $^8A_{2g}$	2.936	299	35226	2.890	272	29714
5 $^8T_{2g}$	2.936	298	35275	2.890	271	29839
6 $^8T_{1g}$	2.937	299	35396	2.890	272	30077
			$4f^9$			
$4f^9(^6H)$						
1 $^6T_{1u}$	2.894	297	15462	2.858	276	11526
1 $^6T_{2u}$	2.894	297	15544	2.856	276	11579
2 $^6T_{1u}$	2.894	297	15572	2.855	277	11953
1 $^6E_{u}$	2.894	297	15491	2.857	277	12121
$4f^9(^6F)$						
3 $^6T_{1u}$	2.893	297	21392	2.855	275	16352
2 $^6T_{2u}$	2.893	297	21526	2.855	275	16546
1 $^6A_{2u}$	2.893	297	21507	2.856	275	16688
$4f^9(^6P)$						
4 $^6T_{1u}$	2.891	296	43108	2.852	271	34262

[a] The energy difference between the Tb^{3+} $4f^8 - 1^7A_{2g}$ and the Tb^{2+} $4f^85d^1 - 1^8A_{1g}$ at MS-RASPT2 level is 3056 cm^{-1}.

Supplementary Table 3. Spectroscopic constants of the ground and excited states of Tb^{3+} and Tb^{2+} -doped SrS octahedral defects calculated using the relativistic spin-free second order Douglas-Kroll-Hess Hamiltonian with AIMP SrS embedding. The embedded cluster RASSCF results include basic bonding interactions and $4f$–shell radial correlation; the MS-RASPT2 ones include also dynamic correlation of 99 or 98 valence electrons in the Tb $5s, 5p$, S $3s, 3p$, Sr $4p$ closed shells and Tb $4f, 5d, 6s$ open shells. Tb–S bond distances ($d_{\mathrm{Tb-S},e}$ in Å), TbS_6 breathing mode harmonic vibrational frequencies ($\omega_{a_{1g}}$ in cm^{-1}), and minimum-to-minimum energy differences (T_e in cm^{-1}) are given. See Figs. 12, 13 and text for details.

	RASSCF			MS-RASPT2		
term	$d_{\mathrm{Tb-S},e}$	$\omega_{a_{1g}}$	T_e	$d_{\mathrm{Tb-S},e}$	$\omega_{a_{1g}}$	T_e
			Tb^{3+}-doped SrS			
$4f^8(^7F)$ [a]						
$1\ ^7A_{2g}$	2.794	297	0	2.779	284	0
$1\ ^7T_{2g}$	2.794	298	697	2.780	284	540
$1\ ^7T_{1g}$	2.795	298	679	2.780	284	894
$4f^8(^5D)$						
$1\ ^5T_{2g}$	2.794	297	25435	2.777	285	21666
$1\ ^5E_{g}$	2.794	297	25429	2.777	287	21710
$4f^8(^5L,^5G)$						
$2\ ^5T_{2g}$	2.794	297	29623	2.777	285	26203
$2\ ^5E_{g}$	2.794	297	29477	2.778	286	26417
$1\ ^5T_{1g}$	2.794	297	29444	2.777	285	26418
$1\ ^5A_{1g}$	2.793	297	29440	2.777	286	26511
$2\ ^5T_{1g}$	2.794	297	29663	2.776	285	26828
$3\ ^5E_{g}$	2.794	297	29709	2.777	286	26983
$2\ ^5A_{1g}$	2.794	297	30296	2.776	286	26990
$3\ ^5T_{2g}$	2.794	297	29716	2.777	285	27233
$3\ ^5T_{1g}$	2.794	297	30332	2.777	285	27410
$4\ ^5E_{g}$	2.794	297	30283	2.779	287	27489
$4\ ^5T_{2g}$	2.794	297	30211	2.778	285	27522
			Tb^{2+}-doped SrS			
			$4f^8(5d+6s)^1$			
$4f^8(^7F)5dt_{2g}^1$						
Octets						
$1\ ^8A_{1g}$	2.951	270	0	2.932	259	0
$1\ ^8T_{1g}$	2.951	270	931	2.931	258	546
$1\ ^8E_{g}$	2.950	269	1252	2.931	260	1046
$1\ ^8T_{2g}$	2.950	270	1934	2.929	258	1662
$1\ ^8A_{2g}$	2.949	270	2226	2.930	259	2385
$2\ ^8T_{2g}$	2.948	271	4352	2.928	258	3342
$2\ ^8E_{g}$	2.947	271	4462	2.929	258	3510
$2\ ^8T_{1g}$	2.947	271	4361	2.927	258	3511
$3\ ^8T_{1g}$	2.946	272	7384	2.925	257	6100
Sextets						
$1\ ^6A_{2g}$	2.943	273	9539	2.926	258	8462
$1\ ^6T_{2g}$	2.943	273	9887	2.925	258	8792
$1\ ^6T_{1g}$	2.942	273	10140	2.924	257	8961
$1\ ^6A_{1g}$	2.943	273	11070	2.925	258	9017
$1\ ^6E_{g}$	2.943	273	11058	2.925	258	9594

2 $^6T_{1g}$	2.943	273	11088	2.925	258	9696
2 $^6E_{g}$	2.942	273	12908	2.922	257	10768
2 $^6T_{2g}$	2.941	273	12709	2.923	258	11019
3 $^6T_{1g}$	2.941	272	13788	2.922	257	11730
$4f^6(^7F)(5de_g^1 + 6s_{a_{1g}}^1)$						
Octets						
3 $^8T_{2g}$	2.987	276	17374	2.972	258	17534
3 $^8E_{g}$	2.986	275	19948	2.969	264	19868
4 $^8T_{1g}$	2.986	275	20179	2.969	257	20186
4 $^8T_{2g}$	2.986	274	21514	2.970	257	20737
5 $^8T_{1g}$	2.983	273	24847	2.967	254	23895
2 $^8A_{2g}$	3.012	278	32542	2.989	262	26795
5 $^8T_{2g}$	3.012	278	32571	2.990	262	26899
6 $^8T_{1g}$	3.013	278	32667	2.990	262	27101
			$4f^9$			
$4f^9(^6H)$						
1 $^6T_{1u}$	2.968	275	14718	2.949	228	1722
1 $^6T_{2u}$	2.968	276	14809	2.947	242	1796
1 $^6E_{u}$	2.968	275	14743	2.950	305	1978
2 $^6T_{1u}$	2.968	276	14837	2.945	254	2244
$4f^9(^6F)$						
1 $^6A_{2u}$	2.968	275	20764	2.949	282	6575
3 $^6T_{1u}$	2.967	275	20674	2.945	253	6602
2 $^6T_{2u}$	2.968	275	20798	2.947	242	6831
$4f^9(^6P)$						
4 $^6T_{1u}$	2.966	275	42438	2.943	253	24351

[a] The energy difference between the Tb^{3+} $4f^8 - 1^7A_{2g}$ and the Tb^{2+} $4f^85d^1 - 1^8A_{1g}$ at MS-RASPT2 level is 3274 cm^{-1}.

Supplementary Table 4. O_{h} $\Gamma_{6\mathrm{u}}$, $\Gamma_{7\mathrm{u}}$, and $\Gamma_{8\mathrm{u}}$ states derived from the lowest energy multiplets of the $4f^9$ configuration.

					2J				$\Gamma_{6\mathrm{u}}$	$\Gamma_{7\mathrm{u}}$	$\Gamma_{8\mathrm{u}}$
$4f^9(^6H_J)$			5	7	9	11	13	15	5	6	11
$4f^9(^6F_J)$	1	3	5	7	9	11			4	3	7
$4f^9(^6P_J)$		3	5	7					1	2	3

Supplementary Table 5. O_h $\mathit{\Gamma}_{6g}$, $\mathit{\Gamma}_{7g}$, and $\mathit{\Gamma}_{8g}$ states derived from the $^7F_J \times (^2T_{2g} +^2 E_g +^2 A_{1g})$ coupling.

	J	Total			High spin			Low spin		
		$\mathit{\Gamma}_{6g}$	$\mathit{\Gamma}_{7g}$	$\mathit{\Gamma}_{8g}$	$\mathit{\Gamma}_{6g}$	$\mathit{\Gamma}_{7g}$	$\mathit{\Gamma}_{8g}$	$\mathit{\Gamma}_{6g}$	$\mathit{\Gamma}_{7g}$	$\mathit{\Gamma}_{8g}$
$4f^8(^7F_J) \times^2 T_{2g}$										
	6	7	6	13	4	3	7	3	3	6
	5	5	6	11	3	3	6	2	3	5
	4	4	5	9	2	3	5	2	2	4
	3	4	3	7	2	2	4	2	1	3
	2	3	2	5	2	1	3	1	1	2
	1	1	2	3	1	1	2	0	1	1
	0	0	1	1	0	1	1	0	0	0
	$^7F_{6,5,4,3,2,1,0}$	24	25	49	14	14	28	10	11	21
$4f^8(^7F_J) \times^2 E_g$										
	6	4	4	9	2	2	5	2	2	4
	5	4	4	7	2	2	4	2	2	3
	4	3	3	6	2	2	3	1	1	3
	3	2	2	5	1	1	3	1	1	2
	2	2	2	3	1	1	2	1	1	1
	1	1	1	2	1	1	1	0	0	1
	0	0	0	1	0	0	1	0	0	0
	$^7F_{6,5,4,3,2,1,0}$	16	16	33	9	9	19	7	7	14
$4f^8(^7F_J) \times^2 A_{1g}$										
	6	2	3	4	1	2	2	1	1	2
	5	2	1	4	1	1	2	1	0	2
	4	2	1	3	1	0	2	1	1	1
	3	1	2	2	1	1	1	0	1	1
	2	0	1	2	0	1	1	0	0	1
	1	1	0	1	0	0	1	1	0	0
	0	1	0	0	1	0	0	0	0	0
	$^7F_{6,5,4,3,2,1,0}$	9	8	16	5	5	9	4	3	7

Supplementary Table 6. Spectroscopic constants and analyses of the spin-orbit wave functions of the ground and lowest lying excited states of Tb^{3+} and Tb^{2+} -doped CaS octahedral defects. Tb–S bond distances ($d_{\mathrm{Tb-S},e}$ in Å), TbS_6 breathing mode harmonic vibrational frequencies ($\omega_{a_{1g}}$ in cm^{-1}) and minimum-to-minimum energy differences (T_e in cm^{-1}) are given. See Fig. 14, 15 and text for details.

State	$d_{\mathrm{Tb-S},e}$	$\omega_{a_{1g}}$	T_e	weights of terms larger than 10% [a]							
				Tb^{3+}-doped CaS							
$4f^8(^7F_{0-6})$											
1 A_{1g}	2.708	306	0	46.52	1 $^7T_{2g}$	38.32	1 $^7A_{2g}$	11.35	1 $^7T_{1g}$		
1 T_{1g}	2.708	306	38	45.30	1 $^7T_{2g}$	35.88	1 $^7A_{2g}$	15.04	1 $^7T_{1g}$		
1 T_{2g}	2.708	306	80	42.04	1 $^7T_{2g}$	34.07	1 $^7A_{2g}$	20.09	1 $^7T_{1g}$		
1 A_{2g}	2.708	306	333	52.47	1 $^7T_{2g}$	43.69	1 $^7T_{1g}$				
2 T_{2g}	2.708	306	381	55.40	1 $^7T_{1g}$	40.35	1 $^7T_{2g}$				
1 E_g	2.708	306	400	59.96	1 $^7T_{1g}$	36.18	1 $^7T_{2g}$				
2 T_{1g}	2.708	306	2304	66.99	1 $^7T_{2g}$	16.04	1 $^7A_{2g}$	14.75	1 $^7T_{1g}$		
3 T_{2g}	2.708	306	2375	57.22	1 $^7T_{1g}$	30.90	1 $^7A_{2g}$				
2 E_g	2.708	306	2541	52.62	1 $^7T_{2g}$	45.17	1 $^7T_{1g}$				
3 T_{1g}	2.708	306	2565	52.00	1 $^7T_{1g}$	43.63	1 $^7T_{2g}$				
2 A_{1g}	2.708	306	3534	50.80	1 $^7A_{2g}$	32.69	1 $^7T_{1g}$	13.22	1 $^7T_{2g}$		
4 T_{1g}	2.708	306	3656	49.37	1 $^7T_{2g}$	24.92	1 $^7A_{2g}$	22.39	1 $^7T_{1g}$		
3 E_g	2.708	306	3773	75.99	1 $^7T_{2g}$	20.68	1 $^7T_{1g}$				
4 T_{2g}	2.708	306	3965	64.28	1 $^7T_{1g}$	28.09	1 $^7T_{2g}$				
5 T_{1g}	2.708	306	4682	49.96	1 $^7T_{2g}$	34.71	1 $^7T_{1g}$	11.46	1 $^7A_{2g}$		
5 T_{2g}	2.708	306	4694	39.46	1 $^7T_{1g}$	36.95	1 $^7T_{2g}$	19.71	1 $^7A_{2g}$		
2 A_{2g}	2.708	306	4839	52.43	1 $^7T_{1g}$	43.65	1 $^7T_{2g}$				
6 T_{2g}	2.708	306	5343	52.99	1 $^7T_{1g}$	35.47	1 $^7T_{2g}$				
4 E_g	2.708	306	5528	67.11	1 $^7T_{1g}$	28.61	1 $^7T_{2g}$				
6 T_{1g}	2.708	306	5790	54.42	1 $^7T_{1g}$	34.96	1 $^7T_{2g}$				
3 A_{1g}	2.708	306	5964	51.97	1 $^7T_{1g}$	36.10	1 $^7T_{2g}$				
$4f^8(^5D_{4-3})$											
4 A_{1g}	2.704	304	19992	58.46	1 ^5E_g	39.31	1 $^5T_{2g}$				
7 T_{1g}	2.704	304	20004	48.98	1 $^5T_{2g}$	48.69	1 ^5E_g				
5 E_g	2.704	304	20012	55.95	1 $^5T_{2g}$	41.64	1 ^5E_g				
7 T_{2g}	2.704	304	20027	76.67	1 $^5T_{2g}$	20.84	1 ^5E_g				
8 T_{1g}	2.704	304	25488	86.69	1 $^5T_{2g}$						
8 T_{2g}	2.704	304	25508	48.60	1 $^5T_{2g}$	47.60	1 ^5E_g				
3 A_{2g}	2.704	304	25542	96.12	1 ^5E_g						
$4f^8(^5D_{2-0},^5L_{10-6},^5G_{6-2})$											
4 A_{2g}	2.704	302	28341	51.58	2 $^5T_{1g}$	42.28	3 ^5E_g				
9 T_{2g}	2.704	302	28342	51.43	2 $^5T_{1g}$	29.55	3 ^5E_g	15.16	2 $^5A_{1g}$		
6 E_g	2.704	303	28344	51.44	2 $^5T_{1g}$	23.47	2 $^5A_{1g}$	22.09	3 ^5E_g		
10 T_{2g}	2.704	303	28608	41.36	3 $^5T_{2g}$	19.04	2 $^5T_{1g}$	16.07	3 $^5T_{1g}$	10.22	2 $^5A_{1g}$
9 T_{1g}	2.704	304	28615	35.91	3 $^5T_{2g}$	18.30	3 ^5E_g	17.64	3 $^5T_{1g}$	17.10	2 $^5T_{1g}$
5 A_{1g}	2.704	306	28717	46.92	3 $^5T_{2g}$	15.28	3 ^5E_g	14.55	4 ^5E_g	11.40	2 $^5T_{2g}$
				10.55	4 $^5T_{2g}$						
10 T_{1g}	2.704	305	28862	33.33	3 $^5T_{1g}$	21.75	4 ^5E_g	17.32	4 $^5T_{2g}$	15.11	2 $^5T_{1g}$
7 E_g	2.704	306	28948	33.59	3 $^5T_{1g}$	31.05	4 $^5T_{2g}$	28.22	4 ^5E_g		
11 T_{2g}	2.704	305	28985	59.76	4 $^5T_{2g}$	20.43	4 ^5E_g	15.13	3 $^5T_{1g}$		
12 T_{2g}	2.704	302	29429	32.77	2 $^5T_{1g}$	19.78	2 $^5A_{1g}$	19.71	3 $^5T_{2g}$		
11 T_{1g}	2.704	304	29456	31.32	2 $^5T_{1g}$	21.41	3 ^5E_g	15.55	3 $^5T_{1g}$	14.97	3 $^5T_{2g}$
12 T_{1g}	2.704	305	29596	33.44	2 $^5T_{1g}$	28.16	2 $^5T_{2g}$	11.99	3 $^5T_{2g}$	10.01	4 ^5E_g
8 E_g	2.704	304	29599	27.58	1 ^5E_g	20.79	1 $^5T_{2g}$	12.67	2 ^5E_g	10.45	2 $^5T_{2g}$
13 T_{2g}	2.704	304	29604	61.74	1 $^5T_{2g}$	24.96	1 ^5E_g				
9 E_g	2.704	305	29620	22.63	1 ^5E_g	22.22	2 $^5T_{2g}$	15.80	1 $^5T_{2g}$	13.65	3 ^5E_g
13 T_{1g}	2.704	304	29709	38.06	2 $^5T_{2g}$	20.18	4 $^5T_{2g}$	15.21	3 $^5T_{1g}$		
6 A_{1g}	2.704	305	29733	44.16	2 $^5T_{2g}$	32.08	4 ^5E_g	16.54	4 $^5T_{2g}$		

14 T_{2g}	2.704	303	29735	54.33	2 $^5T_{2g}$	19.52	3 $^5T_{1g}$	11.85	4 $^5T_{2g}$		
14 T_{1g}	2.704	303	29837	61.61	2 $^5T_{2g}$	15.26	2 $^5T_{1g}$				
10 E_g	2.704	304	29850	58.00	2 $^5T_{2g}$						
7 A_{1g}	2.704	303	29891	34.73	2 ^5E_g	33.88	2 $^5T_{2g}$	10.77	3 ^5E_g		
15 T_{1g}	2.704	304	29916	24.79	1 $^5T_{1g}$	22.30	2 $^5T_{2g}$	16.99	3 $^5T_{2g}$	12.49	2 ^5E_g
				11.13	4 $^5T_{2g}$						
15 T_{2g}	2.704	303	29938	65.13	2 $^5T_{2g}$	20.29	1 $^5T_{1g}$				
5 A_{2g}	2.704	309	29953	61.97	4 ^5E_g	32.48	3 $^5T_{1g}$				
16 T_{2g}	2.704	304	29971	20.35	2 $^5T_{2g}$	20.04	4 $^5T_{2g}$	16.78	3 $^5T_{2g}$	11.93	3 $^5T_{1g}$
16 T_{1g}	2.704	304	29987	66.12	1 $^5T_{1g}$	14.64	2 ^5E_g	10.02	2 $^5T_{2g}$		
11 E_g	2.704	306	30040	54.24	2 ^5E_g	31.73	1 $^5T_{1g}$				
17 T_{2g}	2.704	304	30041	57.17	1 $^5T_{1g}$	21.46	1 $^5A_{1g}$	11.14	2 ^5E_g		
18 T_{2g}	2.704	305	30051	48.27	2 ^5E_g	20.71	1 $^5T_{1g}$				
6 A_{2g}	2.704	306	30057	48.91	2 ^5E_g	42.33	1 $^5T_{1g}$				
12 E_g	2.704	304	30063	62.76	1 $^5A_{1g}$	20.88	1 $^5T_{1g}$				
17 T_{1g}	2.704	304	30079	51.03	1 $^5T_{1g}$	12.57	2 ^5E_g				
8 A_{1g}	2.704	307	30094	60.04	2 ^5E_g	11.64	3 ^5E_g				
18 T_{1g}	2.704	305	30121	47.52	2 ^5E_g	38.27	1 $^5T_{1g}$				
19 T_{2g}	2.704	304	30148	41.03	1 $^5T_{1g}$	28.28	1 $^5A_{1g}$	20.24	2 $^5T_{2g}$		
7 A_{2g}	2.704	306	30175	53.43	1 $^5T_{1g}$	40.76	2 ^5E_g				
20 T_{2g}	2.704	305	30317	37.58	1 $^5T_{1g}$	34.96	1 $^5A_{1g}$	16.07	2 ^5E_g		
13 E_g	2.704	303	30354	31.98	1 $^5A_{1g}$	30.73	1 $^5T_{1g}$	18.35	2 ^5E_g		
9 A_{1g}	2.704	306	30448	57.73	3 ^5E_g	37.09	3 $^5T_{2g}$				
19 T_{1g}	2.704	304	30449	34.91	2 $^5T_{1g}$	28.33	3 $^5T_{2g}$	22.24	3 $^5T_{1g}$		
14 E_g	2.704	304	30549	34.78	3 $^5T_{2g}$	23.88	2 $^5A_{1g}$	12.65	3 $^5T_{1g}$	12.36	3 ^5E_g
21 T_{2g}	2.704	308	30562	24.52	2 $^5T_{1g}$	20.31	3 ^5E_g	16.11	2 $^5A_{1g}$	15.72	4 ^5E_g
				11.16	3 $^5T_{2g}$						
20 T_{1g}	2.704	305	30714	38.45	4 $^5T_{2g}$	21.40	3 ^5E_g	19.52	2 $^5T_{1g}$	15.37	4 ^5E_g
15 E_g	2.704	304	30760	29.85	4 $^5T_{2g}$	29.28	3 $^5T_{1g}$	21.27	3 $^5T_{2g}$	10.23	3 ^5E_g
22 T_{2g}	2.704	305	30834	43.55	3 $^5T_{1g}$	21.80	4 $^5T_{2g}$	19.94	4 ^5E_g		
21 T_{1g}	2.704	305	31258	35.36	3 $^5T_{2g}$	23.34	3 ^5E_g	18.22	2 $^5T_{1g}$	14.07	4 $^5T_{2g}$
23 T_{2g}	2.704	304	31315	27.00	3 $^5T_{2g}$	21.28	2 $^5A_{1g}$	21.18	3 $^5T_{1g}$	17.83	2 $^5T_{1g}$
				10.92	4 ^5E_g						
8 A_{2g}	2.704	307	31357	40.93	3 ^5E_g	29.61	2 $^5T_{1g}$	20.03	3 $^5T_{1g}$		
24 T_{2g}	2.704	304	31474	37.49	4 $^5T_{2g}$	22.17	2 $^5T_{1g}$	19.79	3 ^5E_g	10.13	3 $^5T_{1g}$
16 E_g	2.704	306	31545	41.84	4 ^5E_g	20.12	4 $^5T_{2g}$	19.87	2 $^5T_{1g}$		
22 T_{1g}	2.704	305	31630	45.06	3 $^5T_{1g}$	25.24	4 $^5T_{2g}$	16.37	3 $^5T_{2g}$	12.54	4 ^5E_g
17 E_g	2.704	306	31938	23.81	3 $^5T_{2g}$	19.53	2 $^5T_{1g}$	14.39	3 ^5E_g	12.64	3 $^5T_{1g}$
				12.04	4 ^5E_g	12.02	2 $^5A_{1g}$				
25 T_{2g}	2.704	304	31960	39.34	3 $^5T_{2g}$	18.20	2 $^5T_{1g}$	13.33	3 ^5E_g	11.77	4 $^5T_{2g}$
9 A_{2g}	2.704	305	32061	45.39	3 $^5T_{1g}$	25.15	4 ^5E_g	17.58	2 $^5T_{1g}$	11.62	3 ^5E_g
26 T_{2g}	2.704	305	32302	35.63	3 $^5T_{1g}$	23.48	3 $^5T_{2g}$	23.41	4 $^5T_{2g}$	16.15	4 ^5E_g
23 T_{1g}	2.704	305	32350	31.57	4 $^5T_{2g}$	17.96	3 $^5T_{1g}$	16.48	4 ^5E_g	15.30	1 $^5T_{2g}$
				10.28	1 ^5E_g						
10 A_{1g}	2.704	307	32425	63.53	4 $^5T_{2g}$	35.63	4 ^5E_g				
24 T_{1g}	2.704	305	32443	41.73	1 $^5T_{2g}$	28.25	1 ^5E_g	13.12	4 $^5T_{2g}$		
11 A_{1g}	2.704	304	33799	56.73	1 $^5T_{2g}$	38.19	1 ^5E_g				

Tb^{2+}-doped CaS

$4f^9$

$4f^9(^6H_{15/2,13/2,11/2,9/2,7/2,5/2})$ [b]											
1 Γ_{7u}	2.857	276	9595	63.54	1 $^6T_{1u}$	33.76	1 $^6T_{2u}$				
1 Γ_{8u}	2.857	276	9601	49.31	1 $^6T_{2u}$	48.64	1 $^6T_{1u}$				
1 Γ_{6u}	2.857	277	9796	53.70	1 $^6T_{1u}$	28.51	1 ^6E_u	13.88	2 $^6T_{1u}$		
2 Γ_{8u}	2.857	277	9870	32.98	1 $^6T_{2u}$	30.57	2 $^6T_{1u}$	28.07	1 ^6E_u		
3 Γ_{8u}	2.856	277	9936	62.33	2 $^6T_{1u}$	21.57	1 ^6E_u	14.84	1 $^6T_{2u}$		
4 Γ_{8u}	2.857	276	12037	36.94	1 $^6T_{2u}$	33.52	1 $^6T_{1u}$	16.71	1 ^6E_u	12.68	2 $^6T_{1u}$
2 Γ_{6u}	2.857	276	12092	33.29	2 $^6T_{1u}$	29.92	1 $^6T_{2u}$	29.27	1 $^6T_{1u}$		
5 Γ_{8u}	2.857	277	12104	41.74	2 $^6T_{1u}$	27.32	1 $^6T_{2u}$	24.79	1 $^6T_{1u}$		
3 Γ_{6u}	2.857	277	12130	41.26	2 $^6T_{1u}$	40.81	1 $^6T_{1u}$	17.86	1 ^6E_u		

2 Γ_{7u}	2.857	277	12212	47.12	1 ^6E_u	36.80	1 $^6T_{2u}$				
3 Γ_{7u}	2.857	277	14030	42.58	1 $^6T_{2u}$	23.47	1 $^6T_{1u}$	19.08	1 ^6E_u	13.55	2 $^6T_{1u}$
6 Γ_{8u}	2.856	276	14055	48.51	1 $^6T_{2u}$	31.01	2 $^6T_{1u}$	17.62	1 $^6T_{1u}$		
4 Γ_{6u}	2.857	277	14081	47.33	1 $^6T_{1u}$	43.25	2 $^6T_{1u}$				
7 Γ_{8u}	2.857	276	14185	38.16	1 ^6E_u	30.42	1 $^6T_{1u}$	20.70	2 $^6T_{1u}$	10.52	1 $^6T_{2u}$
8 Γ_{8u}	2.857	276	15438	27.66	1 $^6T_{2u}$	22.58	1 $^6T_{1u}$	15.00	3 $^6T_{1u}$	14.92	2 $^6T_{2u}$
9 Γ_{8u}	2.857	277	15641	33.64	1 $^6T_{1u}$	33.04	3 $^6T_{1u}$	11.64	2 $^6T_{1u}$		
5 Γ_{6u}	2.855	276	15731	40.63	3 $^6T_{1u}$	37.61	2 $^6T_{2u}$	13.93	1 $^6T_{2u}$		
4 Γ_{7u}	2.855	276	15743	35.87	1 $^6T_{2u}$	35.61	2 $^6T_{1u}$	12.12	1 ^6E_u		
10 Γ_{8u}	2.856	276	15985	24.46	1 ^6E_u	24.03	3 $^6T_{1u}$	20.34	2 $^6T_{1u}$	12.39	2 $^6T_{2u}$
5 Γ_{7u}	2.855	276	16200	42.92	2 $^6T_{2u}$	20.17	1 $^6A_{2u}$	15.50	3 $^6T_{1u}$	12.26	1 $^6T_{2u}$
11 Γ_{8u}	2.855	276	16356	31.95	2 $^6T_{2u}$	20.29	3 $^6T_{1u}$	14.68	1 $^6A_{2u}$	11.53	2 $^6T_{1u}$
6 Γ_{7u}	2.855	276	17141	38.26	2 $^6T_{1u}$	29.53	1 $^6T_{2u}$	15.15	2 $^6T_{2u}$		
$4f^9(^6F_{11/2,9/2,7/2,5/2,3/2,1/2})$ [b]											
12 Γ_{8u}	2.857	277	17329	28.26	1 $^6T_{1u}$	24.50	2 $^6T_{1u}$	23.53	1 ^6E_u		
6 Γ_{6u}	2.856	276	17401	37.28	1 $^6T_{2u}$	18.08	2 $^6T_{1u}$	16.98	1 $^6T_{1u}$	11.40	3 $^6T_{1u}$
13 Γ_{8u}	2.855	275	17738	55.26	3 $^6T_{1u}$	42.02	2 $^6T_{2u}$				
14 Γ_{8u}	2.855	274	17839	49.73	3 $^6T_{1u}$	24.08	1 $^6A_{2u}$	17.14	2 $^6T_{2u}$		
7 Γ_{7u}	2.855	276	18023	66.38	2 $^6T_{2u}$						
15 Γ_{8u}	2.856	276	18414	26.95	1 $^6T_{2u}$	26.64	1 $^6T_{1u}$	21.15	2 $^6T_{1u}$	15.25	1 ^6E_u
7 Γ_{6u}	2.856	276	18507	46.74	2 $^6T_{1u}$	30.14	1 ^6E_u	12.86	1 $^6T_{2u}$		
8 Γ_{6u}	2.855	275	19021	86.37	3 $^6T_{1u}$	13.24	2 $^6T_{2u}$				
16 Γ_{8u}	2.855	275	19155	70.93	2 $^6T_{2u}$	22.81	3 $^6T_{1u}$				
8 Γ_{7u}	2.855	275	19196	42.97	1 $^6A_{2u}$	34.87	3 $^6T_{1u}$	21.92	2 $^6T_{2u}$		
9 Γ_{6u}	2.855	275	20138	59.11	3 $^6T_{1u}$	40.42	2 $^6T_{2u}$				
17 Γ_{8u}	2.855	275	20217	45.05	2 $^6T_{2u}$	35.28	3 $^6T_{1u}$	19.30	1 $^6A_{2u}$		
18 Γ_{8u}	2.855	275	20969	45.43	2 $^6T_{2u}$	34.08	3 $^6T_{1u}$	20.09	1 $^6A_{2u}$		
9 Γ_{7u}	2.855	275	21423	44.34	2 $^6T_{2u}$	37.80	3 $^6T_{1u}$	17.40	1 $^6A_{2u}$		
					$4f^85d^1$						
$4f^8(^7F_J)5dt^1_{2g}$ High-Spin coupling [c]											
1 Γ_{7g}	2.833	277	0	46.64	1 $^8A_{1g}$	43.15	1 $^8T_{1g}$				
1 Γ_{8g}	2.833	277	52	46.48	1 $^8A_{1g}$	43.10	1 $^8T_{1g}$				
1 Γ_{6g}	2.833	277	100	48.21	1 $^8A_{1g}$	42.48	1 $^8T_{1g}$				
2 Γ_{6g}	2.833	277	504	55.57	1 $^8T_{1g}$	25.40	1 $^8T_{2g}$				
2 Γ_{8g}	2.833	277	506	48.67	1 $^8T_{1g}$	21.69	1 ^8E_g	21.58	1 $^8T_{2g}$		
3 Γ_{8g}	2.833	277	631	39.25	1 $^8T_{2g}$	36.61	1 ^8E_g	10.51	1 $^8T_{1g}$		
2 Γ_{7g}	2.833	277	682	44.91	1 $^8T_{2g}$	34.23	1 ^8E_g	16.78	1 $^8A_{2g}$		
4 Γ_{8g}	2.833	277	685	48.93	1 $^8T_{1g}$	26.49	1 ^8E_g	15.94	1 $^8T_{2g}$		
5 Γ_{8g}	2.833	277	1611	34.11	1 ^8E_g	29.08	1 $^8T_{2g}$	19.54	1 $^8T_{1g}$	10.02	2 $^8T_{2g}$
3 Γ_{6g}	2.833	277	1725	41.99	1 ^8E_g	22.41	1 $^8T_{2g}$	11.50	1 $^8T_{1g}$		
3 Γ_{7g}	2.833	277	2045	52.80	1 $^8T_{1g}$	14.61	1 ^8E_g	11.83	2 $^8T_{1g}$		
4 Γ_{6g}	2.833	277	2068	25.41	1 $^8A_{2g}$	20.41	1 $^8T_{2g}$	16.62	1 ^8E_g	11.57	1 $^8T_{1g}$
6 Γ_{8g}	2.833	277	2090	49.82	1 $^8T_{1g}$	13.82	1 $^8T_{2g}$				
7 Γ_{8g}	2.833	277	2237	28.67	1 $^8T_{2g}$	17.95	1 $^8T_{1g}$	14.45	1 $^8A_{1g}$	13.40	1 ^8E_g
4 Γ_{7g}	2.833	277	2430	54.82	1 $^8T_{2g}$	24.62	1 $^8A_{1g}$	11.56	1 $^8T_{1g}$		
8 Γ_{8g}	2.833	277	2731	33.00	1 $^8T_{1g}$	27.62	1 ^8E_g	10.75	1 $^8T_{2g}$	10.53	2 $^8T_{2g}$
5 Γ_{6g}	2.832	276	2824	52.25	2 $^8T_{2g}$	16.24	1 $^8A_{1g}$				
9 Γ_{8g}	2.831	277	2906	35.00	2 $^8T_{2g}$	29.60	2 $^8T_{1g}$	18.76	2 ^8E_g	11.08	1 $^8T_{1g}$
5 Γ_{7g}	2.831	277	2961	43.63	2 $^8T_{1g}$	22.30	2 $^8T_{2g}$	16.44	2 ^8E_g	13.15	1 $^8T_{1g}$
6 Γ_{6g}	2.832	277	3062	30.07	1 $^8T_{2g}$	25.33	2 ^8E_g	16.82	2 $^8T_{1g}$	10.29	1 $^8T_{1g}$
10 Γ_{8g}	2.831	277	3256	37.51	2 ^8E_g	33.65	2 $^8T_{1g}$				
11 Γ_{8g}	2.832	275	3515	42.06	1 $^8T_{2g}$	13.56	1 $^8T_{1g}$	11.95	2 $^8T_{2g}$		
12 Γ_{8g}	2.831	279	3539	42.96	2 $^8T_{2g}$	21.96	2 $^8T_{1g}$	13.99	1 $^8T_{1g}$		
6 Γ_{7g}	2.833	277	3728	25.96	1 $^8A_{2g}$	20.36	1 ^8E_g	17.53	1 $^8T_{1g}$	13.74	1 $^8A_{1g}$
				10.53	2 $^8T_{2g}$						
7 Γ_{6g}	2.831	277	3886	44.76	2 $^8T_{2g}$	16.07	3 $^8T_{1g}$	13.18	1 $^8T_{1g}$	12.57	2 $^8T_{1g}$
13 Γ_{8g}	2.832	277	3917	25.62	2 ^8E_g	25.51	1 ^8E_g	20.00	1 $^8T_{2g}$		
7 Γ_{7g}	2.833	277	4001	30.96	1 $^8T_{2g}$	24.60	1 $^8T_{1g}$	11.88	1 ^8E_g		
14 Γ_{8g}	2.833	277	4178	23.85	1 $^8T_{1g}$	21.82	1 ^8E_g	18.68	2 $^8T_{1g}$	17.58	1 $^8T_{2g}$

8 Γ_{7g}	2.831	277	4291	66.54	2 $^8T_{1g}$	15.10	2 $^8T_{2g}$				
15 Γ_{8g}	2.831	277	4481	24.44	2 $^8T_{1g}$	20.90	2 $^8T_{2g}$	18.12	1 $^8T_{2g}$	15.40	2 ^8E_g
				11.96	1 ^8E_g						
8 Γ_{6g}	2.831	277	4607	39.98	2 $^8T_{2g}$	19.06	1 $^8T_{1g}$	12.45	2 $^8T_{1g}$		
16 Γ_{8g}	2.832	277	4608	25.52	1 $^8T_{2g}$	22.20	1 $^8T_{1g}$	12.98	1 ^8E_g	10.14	2 ^8E_g
				10.03	2 $^8T_{2g}$						
9 Γ_{6g}	2.833	277	5001	39.73	1 $^8T_{2g}$	21.35	1 $^8T_{1g}$	20.04	1 ^8E_g		
17 Γ_{8g}	2.831	277	5058	29.64	1 $^8T_{2g}$	18.73	2 $^8T_{2g}$	17.38	2 ^8E_g	11.22	3 $^8T_{1g}$
9 Γ_{7g}	2.831	277	5186	58.12	2 $^8T_{2g}$	20.44	2 $^8T_{1g}$	12.06	1 $^8T_{1g}$		
18 Γ_{8g}	2.831	277	5300	30.17	2 ^8E_g	20.92	2 $^8T_{2g}$	14.78	1 $^8T_{2g}$	10.56	3 $^8T_{1g}$
19 Γ_{8g}	2.830	277	5655	39.92	2 $^8T_{1g}$	23.90	2 $^8T_{2g}$	12.11	3 $^8T_{1g}$	11.97	2 ^8E_g
10 Γ_{7g}	2.831	278	5657	52.63	2 ^8E_g	20.31	2 $^8T_{1g}$				
10 Γ_{6g}	2.831	277	5819	28.68	1 $^8A_{2g}$	21.25	2 $^8T_{1g}$	19.17	1 $^8T_{2g}$	17.68	3 $^8T_{1g}$
20 Γ_{8g}	2.830	276	5913	34.55	3 $^8T_{1g}$	16.15	2 $^8T_{1g}$	14.55	1 $^8A_{2g}$	11.52	1 $^8T_{2g}$
21 Γ_{8g}	2.831	278	5998	27.05	2 $^8T_{2g}$	18.37	2 $^8T_{1g}$	14.74	1 $^8T_{2g}$	11.21	3 $^8T_{1g}$
				10.56	1 $^8A_{2g}$						
11 Γ_{6g}	2.831	277	6370	41.86	2 $^8T_{1g}$	18.77	1 $^8T_{2g}$	15.17	2 ^8E_g	14.22	1 $^8A_{2g}$
11 Γ_{7g}	2.831	277	6450	23.77	1 $^8T_{2g}$	22.56	2 $^8T_{2g}$	18.48	1 $^8A_{2g}$	14.02	3 $^8T_{1g}$
22 Γ_{8g}	2.830	277	6636	26.11	3 $^8T_{1g}$	17.71	2 $^8T_{1g}$	12.82	1 $^8T_{2g}$	11.84	2 $^8T_{2g}$
				11.26	2 ^8E_g	10.47	1 $^8A_{2g}$				
12 Γ_{6g}	2.830	277	6834	65.74	2 $^8T_{1g}$	16.95	2 ^8E_g				
12 Γ_{7g}	2.831	277	6865	32.17	2 $^8T_{2g}$	17.48	1 $^8T_{2g}$	10.82	2 ^8E_g	10.67	1 $^8A_{2g}$
23 Γ_{8g}	2.829	277	6978	53.06	3 $^8T_{1g}$	24.46	2 $^8T_{2g}$				
13 Γ_{6g}	2.829	277	6982	53.54	3 $^8T_{1g}$	22.31	2 $^8T_{2g}$				
24 Γ_{8g}	2.830	277	7096	45.03	2 $^8T_{2g}$	15.26	3 $^8T_{1g}$	13.67	2 $^8T_{1g}$	10.60	2 ^8E_g
25 Γ_{8g}	2.831	277	7324	40.38	2 $^8T_{1g}$	30.76	2 $^8T_{2g}$	20.37	2 ^8E_g		
26 Γ_{8g}	2.830	277	7714	48.98	2 $^8T_{1g}$	28.32	2 ^8E_g	16.18	3 $^8T_{1g}$		
$4f^6(^7F_J)5de_g^1$ Low-Spin coupling [c]											
14 Γ_{6g}	2.827	276	8191	41.69	1 $^6T_{2g}$	25.93	1 $^6A_{2g}$	22.88	1 $^6T_{1g}$		
27 Γ_{8g}	2.827	276	8220	37.83	1 $^6T_{2g}$	23.92	1 $^6T_{1g}$	23.36	1 $^6A_{2g}$		
13 Γ_{7g}	2.827	276	8297	44.18	1 $^6T_{1g}$	31.66	1 $^6T_{2g}$	13.34	1 $^6A_{1g}$		
28 Γ_{8g}	2.827	276	8323	33.71	1 $^6T_{1g}$	28.92	2 $^6T_{1g}$	18.63	1 $^6T_{2g}$	10.33	1 ^6E_g
14 Γ_{7g}	2.829	276	8513	27.80	3 $^8T_{1g}$	26.86	2 $^6T_{1g}$	15.43	1 ^6E_g		
29 Γ_{8g}	2.829	276	8558	24.91	2 $^6T_{1g}$	22.82	3 $^8T_{1g}$	16.71	1 $^6A_{1g}$	13.57	1 ^6E_g
				10.86	1 $^6T_{1g}$						
15 Γ_{6g}	2.829	277	8597	72.47	3 $^8T_{1g}$						
30 Γ_{8g}	2.829	276	8652	41.77	3 $^8T_{1g}$	17.26	2 $^6T_{1g}$	14.91	1 ^6E_g		
15 Γ_{7g}	2.828	276	8719	42.74	3 $^8T_{1g}$	26.98	1 ^6E_g	15.02	2 $^6T_{1g}$		
31 Γ_{8g}	2.826	276	9044	28.63	1 $^6T_{1g}$	23.56	1 $^6T_{2g}$	13.32	2 $^6T_{2g}$		
32 Γ_{8g}	2.827	276	9162	23.56	1 $^6T_{2g}$	19.56	1 $^6A_{2g}$	18.16	1 $^6T_{1g}$	10.87	2 $^6T_{1g}$
				10.27	2 $^6T_{2g}$						
16 Γ_{6g}	2.827	276	9295	50.78	1 $^6T_{2g}$						
16 Γ_{7g}	2.829	275	9721	62.49	3 $^8T_{1g}$	16.97	1 $^6A_{1g}$				
33 Γ_{8g}	2.829	276	9767	77.97	3 $^8T_{1g}$						
17 Γ_{7g}	2.826	277	9883	41.46	1 $^6T_{1g}$	21.79	2 ^6E_g	12.11	3 $^6T_{1g}$	10.94	3 $^8T_{1g}$
34 Γ_{8g}	2.826	276	10063	37.01	1 $^6T_{2g}$	13.30	2 $^6T_{2g}$	12.55	3 $^6T_{1g}$	10.57	2 $^6T_{1g}$
17 Γ_{6g}	2.826	276	10181	27.11	1 $^6A_{2g}$	23.42	2 $^6T_{2g}$	14.25	3 $^6T_{1g}$	14.20	1 $^6T_{2g}$
				11.61	1 $^6T_{1g}$						
35 Γ_{8g}	2.827	276	10250	31.22	1 ^6E_g	31.11	1 $^6T_{1g}$	16.76	2 $^6T_{1g}$		
18 Γ_{7g}	2.828	276	10301	38.84	2 $^6T_{1g}$	18.07	1 $^6A_{1g}$	14.15	3 $^8T_{1g}$	12.80	1 $^6T_{2g}$
18 Γ_{6g}	2.826	276	10838	33.37	1 ^6E_g	18.27	2 ^6E_g	18.22	3 $^6T_{1g}$	11.14	2 $^6T_{1g}$
19 Γ_{6g}	2.826	276	10924	34.24	3 $^6T_{1g}$	18.82	1 $^6T_{1g}$	18.05	2 $^6T_{2g}$		
36 Γ_{8g}	2.827	276	10950	24.69	2 $^6T_{1g}$	20.54	2 $^6T_{2g}$	20.28	2 ^6E_g	10.88	1 $^6A_{1g}$
37 Γ_{8g}	2.826	276	10988	37.67	3 $^6T_{1g}$	17.71	1 $^6T_{2g}$	12.25	2 $^6T_{1g}$	12.10	1 $^6T_{1g}$
38 Γ_{8g}	2.826	276	11202	45.86	2 $^6T_{2g}$	17.70	2 ^6E_g				
19 Γ_{7g}	2.826	276	11246	30.98	3 $^6T_{1g}$	25.28	2 $^6T_{1g}$	20.19	1 $^6T_{2g}$	11.07	1 $^6T_{1g}$
39 Γ_{8g}	2.827	276	11279	37.10	1 $^6T_{2g}$	13.80	2 ^6E_g	13.70	1 $^6A_{2g}$	12.02	2 $^6T_{2g}$
				11.63	3 $^6T_{1g}$						
20 Γ_{7g}	2.826	276	11574	31.29	1 $^6T_{1g}$	29.47	3 $^6T_{1g}$	19.59	2 $^6T_{2g}$		
40 Γ_{8g}	2.827	276	11788	21.12	1 $^6T_{1g}$	19.37	2 $^6T_{2g}$	17.64	2 ^6E_g		
20 Γ_{6g}	2.828	276	11898	28.66	1 $^6T_{2g}$	24.97	2 ^6E_g	16.32	1 ^6E_g	14.69	2 $^6T_{1g}$

41 Γ_{8g}	2.827	276	12263	28.64	1 $^6T_{1g}$	26.68	1 $^6T_{2g}$	14.66	2 $^6T_{2g}$		
21 Γ_{6g}	2.827	276	12423	44.21	2 $^6T_{1g}$	28.97	1 ^6E_g				
42 Γ_{8g}	2.827	276	12440	20.34	1 $^6T_{1g}$	18.30	2 $^6T_{1g}$	14.92	1 $^6T_{2g}$	13.41	3 $^6T_{1g}$
21 Γ_{7g}	2.826	276	12714	35.12	3 $^6T_{1g}$	26.86	2 ^6E_g	13.42	1 $^6T_{1g}$	12.36	2 $^6T_{2g}$
43 Γ_{8g}	2.826	276	12782	28.87	3 $^6T_{1g}$	17.11	1 ^6E_g	15.74	2 $^6T_{2g}$	14.02	2 $^6T_{1g}$
				13.36	2 ^6E_g						
22 Γ_{7g}	2.826	276	12844	26.47	3 $^6T_{1g}$	20.11	2 $^6T_{1g}$	17.47	1 $^6A_{1g}$	17.10	2 $^6T_{2g}$
22 Γ_{6g}	2.827	276	12880	35.94	1 $^6T_{2g}$	20.99	1 $^6T_{1g}$	17.16	2 $^6T_{2g}$	17.08	1 $^6A_{2g}$
23 Γ_{7g}	2.826	276	13406	26.04	2 ^6E_g	24.77	1 $^6T_{1g}$	15.16	1 $^6A_{1g}$	14.83	3 $^6T_{1g}$
44 Γ_{8g}	2.826	276	13416	37.27	2 ^6E_g	21.33	2 $^6T_{2g}$	13.56	3 $^6T_{1g}$		
45 Γ_{8g}	2.827	276	13555	25.01	2 $^6T_{1g}$	15.86	1 ^6E_g	15.00	3 $^6T_{1g}$		
23 Γ_{6g}	2.826	276	13802	40.53	2 $^6T_{2g}$	26.34	3 $^6T_{1g}$	11.16	2 $^6T_{1g}$		
46 Γ_{8g}	2.826	276	13876	26.13	2 $^6T_{2g}$	24.84	2 $^6T_{1g}$	20.50	1 ^6E_g		
47 Γ_{8g}	2.826	276	14245	24.96	3 $^6T_{1g}$	17.46	2 $^6T_{2g}$	16.42	2 $^6T_{1g}$	11.82	2 ^6E_g
				11.60	1 ^6E_g						
24 Γ_{7g}	2.826	276	14590	45.74	2 $^6T_{1g}$	30.17	1 ^6E_g				
24 Γ_{6g}	2.826	276	14764	63.66	2 $^6T_{2g}$	27.08	2 ^6E_g				
48 Γ_{8g}	2.825	276	14859	46.73	3 $^6T_{1g}$	25.32	2 $^6T_{2g}$	13.15	2 ^6E_g		
25 Γ_{7g}	2.825	276	15265	38.43	2 $^6T_{2g}$	37.12	3 $^6T_{1g}$				
49 Γ_{8g}	2.825	276	15887	74.18	3 $^6T_{1g}$	15.98	2 $^6T_{2g}$				

[a] The analyses of the wave functions have been done at d_{Tb-S}= 2.700 Å ($4f^8$), 2.850 Å ($4f^85d^1$), 2.850 Å ($4f^9$).
[b] C.f. Table IV.
[c] C.f. Table V.

Supplementary Table 7. Spectroscopic constants and analyses of the spin-orbit wave functions of the ground and lowest lying excited states of Tb^{3+} and Tb^{2+} -doped SrS octahedral defects. Tb–S bond distances ($d_{\text{Tb-S},e}$ in Å), TbS_6 breathing mode harmonic vibrational frequencies ($\omega_{a_{1g}}$ in cm^{-1}) and minimum-to-minimum energy differences (T_e in cm^{-1}).See Fig. 14, 15 and text for details.

State	$d_{\text{Tb-S},e}$	$\omega_{a_{1g}}$	T_e	weights of terms larger than 10% [a]							
				Tb^{3+}-doped SrS							
$4f^8(^7F_{0-6})$											
1 A_{1g}	2.780	284	0	46.73	1 $^7T_{2g}$	38.00	1 $^7A_{2g}$	11.49	1 $^7T_{1g}$		
1 T_{1g}	2.780	284	35	45.46	1 $^7T_{2g}$	35.51	1 $^7A_{2g}$	15.27	1 $^7T_{1g}$		
1 T_{2g}	2.780	284	74	42.15	1 $^7T_{2g}$	33.64	1 $^7A_{2g}$	20.44	1 $^7T_{1g}$		
1 A_{2g}	2.780	284	302	52.31	1 $^7T_{2g}$	43.89	1 $^7T_{1g}$				
2 T_{2g}	2.780	285	345	55.64	1 $^7T_{1g}$	40.13	1 $^7T_{2g}$				
1 E_g	2.780	285	362	60.20	1 $^7T_{1g}$	35.97	1 $^7T_{2g}$				
2 T_{1g}	2.780	284	2295	66.98	1 $^7T_{2g}$	16.08	1 $^7A_{2g}$	14.73	1 $^7T_{1g}$		
3 T_{2g}	2.780	284	2352	56.97	1 $^7T_{1g}$	31.00	1 $^7A_{2g}$				
2 E_g	2.780	284	2511	52.48	1 $^7T_{2g}$	45.31	1 $^7T_{1g}$				
3 T_{1g}	2.780	284	2532	52.29	1 $^7T_{1g}$	43.37	1 $^7T_{2g}$				
2 A_{1g}	2.780	284	3534	50.98	1 $^7A_{2g}$	32.92	1 $^7T_{1g}$	12.84	1 $^7T_{2g}$		
4 T_{1g}	2.780	284	3650	49.25	1 $^7T_{2g}$	24.88	1 $^7A_{2g}$	22.58	1 $^7T_{1g}$		
3 E_g	2.780	285	3757	76.19	1 $^7T_{2g}$	20.51	1 $^7T_{1g}$				
4 T_{2g}	2.780	284	3931	64.80	1 $^7T_{1g}$	27.72	1 $^7T_{2g}$				
5 T_{1g}	2.780	285	4667	49.99	1 $^7T_{2g}$	34.49	1 $^7T_{1g}$	11.68	1 $^7A_{2g}$		
5 T_{2g}	2.780	284	4680	39.47	1 $^7T_{1g}$	36.84	1 $^7T_{2g}$	19.84	1 $^7A_{2g}$		
2 A_{2g}	2.780	284	4811	52.26	1 $^7T_{1g}$	43.83	1 $^7T_{2g}$				
6 T_{2g}	2.780	285	5319	52.18	1 $^7T_{1g}$	35.93	1 $^7T_{2g}$				
4 E_g	2.780	284	5495	66.95	1 $^7T_{1g}$	28.82	1 $^7T_{2g}$				
6 T_{1g}	2.780	285	5767	54.01	1 $^7T_{1g}$	35.20	1 $^7T_{2g}$				
3 A_{1g}	2.780	285	5943	51.61	1 $^7T_{1g}$	36.31	1 $^7T_{2g}$				
$4f^8(^5D_{4-3})$											
4 A_{1g}	2.777	286	20128	58.40	1 ^5E_g	39.39	1 $^5T_{2g}$				
7 T_{1g}	2.777	286	20138	49.10	1 $^5T_{2g}$	48.57	1 ^5E_g				
5 E_g	2.777	286	20145	56.09	1 $^5T_{2g}$	41.53	1 ^5E_g				
7 T_{2g}	2.778	286	20156	76.82	1 $^5T_{2g}$	20.71	1 ^5E_g				
8 T_{1g}	2.777	285	25625	86.69	1 $^5T_{2g}$						
8 T_{2g}	2.777	286	25646	48.67	1 $^5T_{2g}$	47.56	1 ^5E_g				
3 A_{2g}	2.777	286	25680	96.15	1 ^5E_g						
$4f^8(^5D_{2-0}, ^5L_{10-6}, ^5G_{6-2})$											
4 A_{2g}	2.777	285	28519	51.94	2 $^5T_{1g}$	39.46	3 ^5E_g				
9 T_{2g}	2.776	286	28520	51.85	2 $^5T_{1g}$	29.55	3 ^5E_g	15.00	2 $^5A_{1g}$		
6 E_g	2.777	286	28523	51.90	2 $^5T_{1g}$	23.35	2 $^5A_{1g}$	22.00	3 ^5E_g		
10 T_{2g}	2.777	285	28763	42.23	3 $^5T_{2g}$	18.46	2 $^5T_{1g}$	16.38	3 $^5T_{1g}$		
9 T_{1g}	2.777	285	28767	36.45	3 $^5T_{2g}$	18.21	3 $^5T_{1g}$	17.49	3 ^5E_g	16.89	2 $^5T_{1g}$
5 A_{1g}	2.777	286	28861	47.64	3 $^5T_{2g}$	14.66	3 ^5E_g	14.46	4 ^5E_g	11.54	2 $^5T_{2g}$
				10.49	4 $^5T_{2g}$						
10 T_{1g}	2.778	285	28998	33.15	3 $^5T_{1g}$	21.23	4 ^5E_g	17.38	4 $^5T_{2g}$	15.25	2 $^5T_{1g}$
7 E_g	2.778	286	29075	33.83	3 $^5T_{1g}$	31.37	4 $^5T_{2g}$	27.57	4 ^5E_g		
11 T_{2g}	2.778	286	29106	60.65	4 $^5T_{2g}$	19.72	4 ^5E_g	14.93	3 $^5T_{1g}$		
12 T_{2g}	2.777	285	29594	34.17	2 $^5T_{1g}$	19.83	2 $^5A_{1g}$	18.84	3 $^5T_{2g}$		
11 T_{1g}	2.777	286	29620	32.49	2 $^5T_{1g}$	21.03	3 ^5E_g	15.26	3 $^5T_{1g}$	14.52	3 $^5T_{2g}$
13 T_{2g}	2.777	286	29751	61.70	1 $^5T_{2g}$	25.16	1 ^5E_g				
8 E_g	2.777	286	29753	28.58	1 ^5E_g	21.49	1 $^5T_{2g}$	11.99	2 ^5E_g	10.89	2 $^5T_{2g}$
12 T_{1g}	2.777	285	29758	32.14	2 $^5T_{1g}$	28.77	2 $^5T_{2g}$	12.16	3 $^5T_{2g}$		
9 E_g	2.777	286	29778	24.14	2 $^5T_{2g}$	21.99	1 ^5E_g	15.24	1 $^5T_{2g}$	12.97	3 ^5E_g
13 T_{1g}	2.777	285	29863	36.72	2 $^5T_{2g}$	20.92	4 $^5T_{2g}$	15.29	3 $^5T_{1g}$		
6 A_{1g}	2.777	286	29887	46.83	2 $^5T_{2g}$	30.45	4 ^5E_g	16.34	4 $^5T_{2g}$		

14 T_{2g}	2.777	285	29894	54.18	2 $^5T_{2g}$	19.58	3 $^5T_{1g}$	11.91	4 $^5T_{2g}$		
14 T_{1g}	2.777	285	30000	61.03	2 $^5T_{2g}$	15.42	2 $^5T_{1g}$				
10 E_g	2.777	285	30013	55.24	2 $^5T_{2g}$						
7 A_{1g}	2.777	286	30047	32.86	2 ^5E_g	32.09	2 $^5T_{2g}$	11.47	3 ^5E_g	10.11	4 ^5E_g
15 T_{1g}	2.777	285	30062	25.45	1 $^5T_{1g}$	23.32	2 $^5T_{2g}$	17.23	3 $^5T_{2g}$	11.20	4 $^5T_{2g}$
				10.66	2 ^5E_g						
5 A_{2g}	2.778	286	30087	61.54	4 ^5E_g	33.26	3 $^5T_{1g}$				
15 T_{2g}	2.777	285	30098	64.44	2 $^5T_{2g}$	23.19	1 $^5T_{1g}$				
16 T_{2g}	2.777	286	30112	21.22	2 $^5T_{2g}$	19.95	4 $^5T_{2g}$	17.50	3 $^5T_{2g}$	12.22	3 $^5T_{1g}$
16 T_{1g}	2.777	285	30132	69.33	1 $^5T_{1g}$	12.58	2 ^5E_g				
17 T_{2g}	2.778	286	30178	59.63	1 $^5T_{1g}$	21.45	1 $^5A_{1g}$				
11 E_g	2.777	286	30182	44.17	2 ^5E_g	41.42	1 $^5T_{1g}$				
18 T_{2g}	2.778	286	30185	51.88	2 ^5E_g	24.24	1 $^5T_{1g}$				
6 A_{2g}	2.778	285	30193	49.75	1 $^5T_{1g}$	42.73	2 ^5E_g				
12 E_g	2.777	286	30205	60.39	1 $^5A_{1g}$	19.32	2 ^5E_g	12.25	1 $^5T_{1g}$		
17 T_{1g}	2.778	285	30212	53.40	1 $^5T_{1g}$	10.03	2 $^5T_{2g}$				
8 A_{1g}	2.778	286	30230	62.25	2 ^5E_g	10.29	3 ^5E_g				
18 T_{1g}	2.777	286	30271	54.88	2 ^5E_g	31.17	1 $^5T_{1g}$				
19 T_{2g}	2.777	286	30295	35.48	1 $^5T_{1g}$	30.76	1 $^5A_{1g}$	20.58	2 $^5T_{2g}$		
7 A_{2g}	2.778	286	30321	47.56	2 ^5E_g	45.88	1 $^5T_{1g}$				
20 T_{2g}	2.777	286	30463	37.31	1 $^5A_{1g}$	34.90	1 $^5T_{1g}$	16.86	2 ^5E_g		
13 E_g	2.777	286	30500	33.74	1 $^5A_{1g}$	29.03	1 $^5T_{1g}$	18.97	2 ^5E_g		
19 T_{1g}	2.777	285	30603	35.22	2 $^5T_{1g}$	28.28	3 $^5T_{2g}$	22.04	3 $^5T_{1g}$		
9 A_{1g}	2.777	286	30608	59.63	3 ^5E_g	35.34	3 $^5T_{2g}$				
14 E_g	2.777	286	30704	34.87	3 $^5T_{2g}$	24.86	2 $^5A_{1g}$	13.01	3 ^5E_g	11.82	3 $^5T_{1g}$
21 T_{2g}	2.777	286	30724	23.71	2 $^5T_{1g}$	20.66	3 ^5E_g	15.96	2 $^5A_{1g}$	15.23	4 ^5E_g
				11.50	3 $^5T_{2g}$						
20 T_{1g}	2.777	285	30857	38.74	4 $^5T_{2g}$	21.62	3 ^5E_g	18.82	2 $^5T_{1g}$	15.12	4 ^5E_g
15 E_g	2.778	285	30893	29.64	3 $^5T_{1g}$	29.61	4 $^5T_{2g}$	21.20	3 $^5T_{2g}$	10.42	3 ^5E_g
22 T_{2g}	2.778	286	30968	43.66	3 $^5T_{1g}$	21.49	4 $^5T_{2g}$	20.65	4 ^5E_g		
21 T_{1g}	2.777	286	31414	34.76	3 $^5T_{2g}$	24.26	3 ^5E_g	18.68	2 $^5T_{1g}$	13.47	4 $^5T_{2g}$
23 T_{2g}	2.777	286	31472	27.45	3 $^5T_{2g}$	21.48	2 $^5A_{1g}$	20.50	3 $^5T_{1g}$	17.71	2 $^5T_{1g}$
				11.03	4 ^5E_g						
8 A_{2g}	2.777	286	31529	40.83	3 ^5E_g	28.95	2 $^5T_{1g}$	20.14	3 $^5T_{1g}$		
24 T_{2g}	2.777	285	31621	38.06	4 $^5T_{2g}$	21.49	2 $^5T_{1g}$	19.52	3 ^5E_g	10.00	3 $^5T_{1g}$
16 E_g	2.777	286	31692	42.42	4 ^5E_g	19.73	4 $^5T_{2g}$	19.53	2 $^5T_{1g}$		
22 T_{1g}	2.778	285	31763	45.51	3 $^5T_{1g}$	24.67	4 $^5T_{2g}$	16.26	3 $^5T_{2g}$	12.79	4 ^5E_g
17 E_g	2.777	286	32100	23.16	3 $^5T_{2g}$	19.76	2 $^5T_{1g}$	14.61	3 ^5E_g	12.55	3 $^5T_{1g}$
				12.35	2 $^5A_{1g}$	12.19	4 ^5E_g				
25 T_{2g}	2.777	285	32117	38.31	3 $^5T_{2g}$	19.03	2 $^5T_{1g}$	14.20	3 ^5E_g	10.86	4 $^5T_{2g}$
9 A_{2g}	2.777	286	32210	44.44	3 $^5T_{1g}$	25.23	4 ^5E_g	17.95	2 $^5T_{1g}$	12.11	3 ^5E_g
26 T_{2g}	2.777	286	32437	35.60	3 $^5T_{1g}$	23.17	4 $^5T_{2g}$	23.16	3 $^5T_{2g}$	16.69	4 ^5E_g
23 T_{1g}	2.777	286	32489	32.22	4 $^5T_{2g}$	18.01	3 $^5T_{1g}$	17.38	4 ^5E_g	14.36	1 $^5T_{2g}$
10 A_{1g}	2.778	286	32552	62.91	4 $^5T_{2g}$	36.29	4 ^5E_g				
24 T_{1g}	2.777	286	32577	42.50	1 $^5T_{2g}$	28.89	1 ^5E_g	12.34	4 $^5T_{2g}$		
11 A_{1g}	2.777	286	33943	56.68	1 $^5T_{2g}$	38.28	1 ^5E_g				

Tb^{2+}-doped SrS

$4f^9$

$4f^9(^6H_{15/2,13/2,11/2,9/2,7/2,5/2})$ [b]											
1 Γ_{7u}	2.948	233	0	63.22	1 $^6T_{1u}$	33.35	1 $^6T_{2u}$				
1 Γ_{8u}	2.948	232	5	51.48	1 $^6T_{1u}$	45.30	1 $^6T_{2u}$				
1 Γ_{6u}	2.949	253	87	51.65	1 $^6T_{1u}$	31.99	1 ^6E_u	12.87	2 $^6T_{1u}$		
2 Γ_{8u}	2.948	253	187	34.14	1 $^6T_{2u}$	31.19	1 ^6E_u	29.15	2 $^6T_{1u}$		
3 Γ_{8u}	2.947	251	298	58.20	2 $^6T_{1u}$	25.02	1 ^6E_u	15.47	1 $^6T_{2u}$		
4 Γ_{8u}	2.948	244	2384	38.87	1 $^6T_{2u}$	34.65	1 $^6T_{1u}$	16.67	1 ^6E_u		
2 Γ_{7u}	2.949	266	2436	53.31	1 ^6E_u	33.51	1 $^6T_{2u}$				
2 Γ_{6u}	2.948	247	2445	40.88	1 $^6T_{1u}$	24.00	1 ^6E_u	20.81	2 $^6T_{1u}$	14.11	1 $^6T_{2u}$
5 Γ_{8u}	2.947	243	2511	44.12	2 $^6T_{1u}$	26.00	1 $^6T_{2u}$	23.57	1 $^6T_{1u}$		
3 Γ_{6u}	2.947	243	2554	52.18	2 $^6T_{1u}$	30.12	1 $^6T_{1u}$	16.55	1 $^6T_{2u}$		

3 Γ_{7u}	2.948	245	4358		42.80	$1\,^6T_{2u}$	26.16	$1\,^6T_{1u}$	17.50	$1\,^6E_{u}$	11.86	$2\,^6T_{1u}$
6 Γ_{8u}	2.947	246	4458		38.11	$1\,^6E_{u}$	30.40	$1\,^6T_{1u}$	21.23	$2\,^6T_{1u}$		
7 Γ_{8u}	2.948	250	4470		50.10	$1\,^6T_{2u}$	30.51	$2\,^6T_{1u}$	15.04	$1\,^6T_{1u}$		
4 Γ_{6u}	2.948	240	4479		49.50	$1\,^6T_{1u}$	41.57	$2\,^6T_{1u}$				
8 Γ_{8u}	2.948	242	5759		27.65	$1\,^6T_{2u}$	24.52	$1\,^6T_{1u}$	14.15	$2\,^6T_{2u}$	13.34	$3\,^6T_{1u}$
9 Γ_{8u}	2.948	243	5981		36.37	$1\,^6T_{1u}$	30.13	$3\,^6T_{1u}$	12.85	$2\,^6T_{1u}$		
4 Γ_{7u}	2.946	250	6093		38.74	$1\,^6T_{2u}$	33.15	$2\,^6T_{1u}$	10.67	$1\,^6E_{u}$		
5 Γ_{6u}	2.946	246	6095		38.35	$3\,^6T_{1u}$	35.10	$2\,^6T_{2u}$	16.95	$1\,^6T_{2u}$		
10 Γ_{8u}	2.948	251	6318		24.18	$3\,^6T_{1u}$	23.90	$1\,^6E_{u}$	19.98	$2\,^6T_{1u}$	12.50	$2\,^6T_{2u}$
5 Γ_{7u}	2.947	251	6544		39.04	$2\,^6T_{2u}$	22.95	$1\,^6A_{2u}$	15.37	$1\,^6T_{2u}$	13.38	$3\,^6T_{1u}$
11 Γ_{8u}	2.947	250	6759		30.14	$2\,^6T_{2u}$	18.95	$1\,^6A_{2u}$	17.27	$3\,^6T_{1u}$	12.76	$2\,^6T_{1u}$
6 Γ_{7u}	2.946	250	7522		42.37	$2\,^6T_{1u}$	26.34	$1\,^6T_{2u}$	14.93	$2\,^6T_{2u}$		
$4f^9(^6F_{11/2,9/2,7/2,5/2,3/2,1/2})$ [b]												
12 Γ_{8u}	2.948	252	7664		27.54	$2\,^6T_{1u}$	24.35	$1\,^6T_{1u}$	19.20	$1\,^6E_{u}$	11.87	$1\,^6A_{2u}$
6 Γ_{6u}	2.947	246	7863		31.66	$1\,^6T_{2u}$	22.25	$2\,^6T_{1u}$	14.28	$1\,^6T_{1u}$	13.00	$3\,^6T_{1u}$
					10.20	$1\,^6E_{u}$						
13 Γ_{8u}	2.946	251	8179		60.90	$3\,^6T_{1u}$	25.23	$2\,^6T_{2u}$				
14 Γ_{8u}	2.946	249	8230		49.47	$3\,^6T_{1u}$	27.42	$2\,^6T_{2u}$	13.45	$1\,^6A_{2u}$		
7 Γ_{7u}	2.947	247	8488		60.52	$2\,^6T_{2u}$	12.27	$3\,^6T_{1u}$				
15 Γ_{8u}	2.947	245	8834		27.26	$1\,^6T_{2u}$	26.43	$1\,^6T_{1u}$	20.42	$2\,^6T_{1u}$	12.34	$1\,^6E_{u}$
7 Γ_{6u}	2.947	251	8873		45.69	$2\,^6T_{1u}$	25.70	$1\,^6E_{u}$	15.56	$1\,^6T_{2u}$		
8 Γ_{6u}	2.945	254	9458		86.42	$3\,^6T_{1u}$	13.25	$2\,^6T_{2u}$				
8 Γ_{7u}	2.947	253	9499		37.83	$1\,^6A_{2u}$	31.24	$3\,^6T_{1u}$	30.56	$2\,^6T_{2u}$		
16 Γ_{8u}	2.946	247	9597		72.88	$2\,^6T_{2u}$	21.02	$3\,^6T_{1u}$				
9 Γ_{6u}	2.946	250	10583		58.14	$3\,^6T_{1u}$	41.29	$2\,^6T_{2u}$				
17 Γ_{8u}	2.947	248	10598		49.58	$2\,^6T_{2u}$	31.34	$3\,^6T_{1u}$	18.64	$1\,^6A_{2u}$		
18 Γ_{8u}	2.946	250	11355		45.91	$2\,^6T_{2u}$	38.76	$3\,^6T_{1u}$	14.77	$1\,^6A_{2u}$		
9 Γ_{7u}	2.946	249	11813		45.04	$2\,^6T_{2u}$	40.01	$3\,^6T_{1u}$	14.31	$1\,^6A_{2u}$		
						$4f^8 5d^1$						
$4f^8(^7F_J)5dt_{2g}^1$ High-Spin coupling [c]												
1 Γ_{7g}	2.931	259	205		47.39	$1\,^8A_{1g}$	42.71	$1\,^8T_{1g}$				
1 Γ_{8g}	2.931	259	257	1.00	47.26	$1\,^8A_{1g}$	42.67	$1\,^8T_{1g}$				
1 Γ_{6g}	2.931	259	305	0.01	48.94	$1\,^8A_{1g}$	42.02	$1\,^8T_{1g}$				
2 Γ_{8g}	2.931	258	718	1.26	49.09	$1\,^8T_{1g}$	21.99	$1\,^8E_{g}$	20.91	$1\,^8T_{2g}$		
2 Γ_{6g}	2.931	258	719	0.08	56.51	$1\,^8T_{1g}$	24.54	$1\,^8T_{2g}$				
3 Γ_{8g}	2.931	259	843	1.00	38.45	$1\,^8T_{2g}$	36.97	$1\,^8E_{g}$	11.32	$1\,^8T_{1g}$		
2 Γ_{7g}	2.931	259	894		44.54	$1\,^8T_{2g}$	34.88	$1\,^8E_{g}$	16.53	$1\,^8A_{2g}$		
4 Γ_{8g}	2.931	258	898	0.64	48.96	$1\,^8T_{1g}$	26.57	$1\,^8E_{g}$	15.97	$1\,^8T_{2g}$		
5 Γ_{8g}	2.931	259	1818	1.71	35.42	$1\,^8E_{g}$	28.18	$1\,^8T_{2g}$	19.91	$1\,^8T_{1g}$		
3 Γ_{6g}	2.931	259	1929	0.14	44.01	$1\,^8E_{g}$	21.72	$1\,^8T_{2g}$	11.30	$1\,^8T_{1g}$		
3 Γ_{7g}	2.931	258	2237		54.35	$1\,^8T_{1g}$	14.24	$1\,^8E_{g}$	10.80	$2\,^8T_{1g}$		
4 Γ_{6g}	2.931	258	2277	0.78	26.22	$1\,^8A_{2g}$	21.60	$1\,^8T_{2g}$	15.59	$1\,^8E_{g}$	12.08	$1\,^8T_{1g}$
6 Γ_{8g}	2.931	258	2289	52.07	51.73	$1\,^8T_{1g}$	14.18	$1\,^8T_{2g}$				
7 Γ_{8g}	2.931	258	2449	6.77	27.89	$1\,^8T_{2g}$	17.99	$1\,^8T_{1g}$	15.23	$1\,^8A_{1g}$	14.46	$1\,^8E_{g}$
					10.03	$1\,^8A_{2g}$						
4 Γ_{7g}	2.931	258	2653		53.24	$1\,^8T_{2g}$	24.80	$1\,^8A_{1g}$	12.76	$1\,^8T_{1g}$		
8 Γ_{8g}	2.931	258	2934	1.92	33.21	$1\,^8T_{1g}$	28.12	$1\,^8E_{g}$	11.87	$1\,^8T_{2g}$		
5 Γ_{6g}	2.929	257	3097	0.07	49.23	$2\,^8T_{2g}$	19.49	$1\,^8A_{1g}$	11.53	$1\,^8T_{2g}$		
9 Γ_{8g}	2.929	258	3221	37.95	37.99	$2\,^8T_{2g}$	26.05	$2\,^8T_{1g}$	18.07	$2\,^8E_{g}$	11.29	$1\,^8T_{1g}$
5 Γ_{7g}	2.928	257	3298	0.01	40.81	$2\,^8T_{1g}$	23.34	$2\,^8T_{2g}$	16.62	$2\,^8E_{g}$	13.42	$1\,^8T_{1g}$
6 Γ_{6g}	2.929	259	3312	1.34	29.18	$2\,^8E_{g}$	24.16	$1\,^8T_{2g}$	20.25	$2\,^8T_{1g}$		
10 Γ_{8g}	2.929	258	3587	2.03	38.53	$2\,^8E_{g}$	29.45	$2\,^8T_{1g}$				
11 Γ_{8g}	2.930	258	3753	1.37	39.41	$1\,^8T_{2g}$	14.17	$1\,^8T_{1g}$	11.57	$2\,^8T_{1g}$		
12 Γ_{8g}	2.929	258	3855	12.92	41.45	$2\,^8T_{2g}$	22.56	$2\,^8T_{1g}$	13.72	$1\,^8T_{1g}$		
6 Γ_{7g}	2.931	259	3945		25.79	$1\,^8A_{2g}$	20.05	$1\,^8E_{g}$	19.21	$1\,^8T_{1g}$	13.73	$1\,^8A_{1g}$
13 Γ_{8g}	2.931	258	4165	3.58	26.56	$1\,^8E_{g}$	22.68	$2\,^8E_{g}$	21.83	$1\,^8T_{2g}$		
7 Γ_{6g}	2.928	258	4207	1.58	45.71	$2\,^8T_{2g}$	14.46	$3\,^8T_{1g}$	13.10	$1\,^8T_{1g}$	13.06	$2\,^8T_{1g}$
7 Γ_{7g}	2.931	258	4213		33.40	$1\,^8T_{2g}$	22.98	$1\,^8T_{1g}$	12.63	$1\,^8E_{g}$		
14 Γ_{8g}	2.931	258	4400	0.10	22.95	$1\,^8E_{g}$	22.26	$1\,^8T_{1g}$	21.35	$1\,^8T_{2g}$	17.12	$2\,^8T_{1g}$

$8\ \Gamma_{7g}$	2.928	258	4628		67.51	$2\,{}^{8}T_{1g}$	17.65	$2\,{}^{8}T_{2g}$				
$15\ \Gamma_{8g}$	2.929	257	4776	19.05	25.08	$1\,{}^{8}T_{2g}$	23.84	$2\,{}^{8}T_{1g}$	15.14	$2\,{}^{8}E_{g}$	13.60	$2\,{}^{8}T_{2g}$
					13.26	$1\,{}^{8}E_{g}$						
$16\ \Gamma_{8g}$	2.930	259	4856	3.46	19.53	$1\,{}^{8}T_{1g}$	19.51	$1\,{}^{8}T_{2g}$	18.56	$2\,{}^{8}T_{2g}$	11.36	$1\,{}^{8}E_{g}$
$8\ \Gamma_{6g}$	2.929	258	4911	1.95	40.39	$2\,{}^{8}T_{2g}$	19.67	$1\,{}^{8}T_{1g}$	12.78	$2\,{}^{8}T_{1g}$		
$9\ \Gamma_{6g}$	2.931	258	5217	0.44	42.11	$1\,{}^{8}T_{2g}$	21.05	$1\,{}^{8}E_{g}$	19.61	$1\,{}^{8}T_{1g}$		
$17\ \Gamma_{8g}$	2.928	258	5387	18.84	26.33	$1\,{}^{8}T_{2g}$	19.77	$2\,{}^{8}E_{g}$	19.56	$2\,{}^{8}T_{2g}$	13.22	$2\,{}^{8}T_{1g}$
$9\ \Gamma_{7g}$	2.928	258	5509		57.83	$2\,{}^{8}T_{2g}$	18.46	$2\,{}^{8}T_{1g}$	12.19	$1\,{}^{8}T_{1g}$		
$18\ \Gamma_{8g}$	2.929	258	5627	7.48	33.01	$2\,{}^{8}E_{g}$	21.15	$2\,{}^{8}T_{2g}$	15.06	$1\,{}^{8}T_{2g}$		
					10.14	$2\,{}^{6}T_{1u}$						
$10\ \Gamma_{7g}$	2.929	258	5965		50.44	$2\,{}^{8}E_{g}$	22.77	$2\,{}^{8}T_{1g}$	10.11	$1\,{}^{8}A_{2g}$		
$19\ \Gamma_{8g}$	2.928	258	6014	4.45	40.93	$2\,{}^{8}T_{1g}$	24.29	$2\,{}^{8}T_{2g}$	12.25	$2\,{}^{8}E_{g}$	11.04	$3\,{}^{8}T_{1g}$
$10\ \Gamma_{6g}$	2.929	258	6118	0.48	33.95	$1\,{}^{8}A_{2g}$	22.69	$1\,{}^{8}T_{2g}$	16.30	$2\,{}^{8}T_{1g}$	15.47	$3\,{}^{8}T_{1g}$
$20\ \Gamma_{8g}$	2.929	257	6244	0.07	26.28	$1\,{}^{8}A_{2g}$	22.14	$1\,{}^{8}T_{2g}$	17.25	$3\,{}^{8}T_{1g}$	12.05	$2\,{}^{8}T_{2g}$
$21\ \Gamma_{8g}$	2.928	259	6325	32.78	27.97	$2\,{}^{8}T_{1g}$	26.99	$3\,{}^{8}T_{1g}$	26.82	$2\,{}^{8}T_{2g}$		
$11\ \Gamma_{6g}$	2.928	258	6709	0.23	47.29	$2\,{}^{8}T_{1g}$	15.70	$1\,{}^{8}T_{2g}$	15.57	$2\,{}^{8}E_{g}$	10.87	$1\,{}^{8}A_{2g}$
$11\ \Gamma_{7g}$	2.928	258	6769		24.92	$1\,{}^{8}T_{2g}$	20.71	$2\,{}^{8}T_{2g}$	19.48	$1\,{}^{8}A_{2g}$	12.29	$3\,{}^{8}T_{1g}$
$22\ \Gamma_{8g}$	2.928	258	6986	18.77	26.07	$3\,{}^{8}T_{1g}$	17.51	$2\,{}^{8}T_{1g}$	14.00	$2\,{}^{8}E_{g}$	12.60	$1\,{}^{8}T_{2g}$
					10.86	$2\,{}^{8}T_{2g}$						
$12\ \Gamma_{7g}$	2.928	258	7190	0.01	35.45	$2\,{}^{8}T_{2g}$	14.84	$1\,{}^{8}T_{2g}$	12.01	$2\,{}^{8}T_{1g}$	10.79	$2\,{}^{8}E_{g}$
$12\ \Gamma_{6g}$	2.928	258	7190	1.00	66.65	$2\,{}^{8}T_{1g}$	17.75	$2\,{}^{8}E_{g}$				
$23\ \Gamma_{8g}$	2.926	258	7372	4.94	55.69	$3\,{}^{8}T_{1g}$	23.71	$2\,{}^{8}T_{2g}$				
$13\ \Gamma_{6g}$	2.926	258	7377	0.02	57.27	$3\,{}^{8}T_{1g}$	21.42	$2\,{}^{8}T_{2g}$				
$24\ \Gamma_{8g}$	2.928	258	7442		44.46	$2\,{}^{8}T_{2g}$	18.32	$3\,{}^{8}T_{1g}$	14.13	$2\,{}^{8}T_{1g}$		
$25\ \Gamma_{8g}$	2.928	258	7662	4.47	43.47	$2\,{}^{8}T_{1g}$	28.62	$2\,{}^{8}T_{2g}$	20.51	$2\,{}^{8}E_{g}$		
$26\ \Gamma_{8g}$	2.928	258	8078	22.43	48.94	$2\,{}^{8}T_{1g}$	28.28	$2\,{}^{8}E_{g}$	16.78	$3\,{}^{8}T_{1g}$		

$4f^6(^7F_J)5de_g^1$ Low-Spin coupling [c]

$14\ \Gamma_{6g}$	2.925	258	8631	128.33	40.72	$1\,{}^{6}T_{2g}$	27.08	$1\,{}^{6}A_{2g}$	21.90	$1\,{}^{6}T_{1g}$		
$27\ \Gamma_{8g}$	2.925	258	8663	955.83	37.42	$1\,{}^{6}T_{2g}$	25.41	$1\,{}^{6}A_{2g}$	20.76	$1\,{}^{6}T_{1g}$		
$28\ \Gamma_{8g}$	2.926	257	8757	100.94	30.88	$1\,{}^{6}T_{1g}$	30.16	$2\,{}^{6}T_{1g}$	18.40	$1\,{}^{6}T_{2g}$	10.43	$1\,{}^{6}E_{g}$
$13\ \Gamma_{7g}$	2.925	257	8757	0.01	40.40	$1\,{}^{6}T_{1g}$	30.06	$1\,{}^{6}T_{2g}$	10.38	$1\,{}^{6}A_{1g}$		
$14\ \Gamma_{7g}$	2.926	258	8902	0.03	24.39	$2\,{}^{6}T_{1g}$	23.30	$3\,{}^{8}T_{1g}$	14.92	$1\,{}^{6}E_{g}$	13.58	$1\,{}^{6}T_{1g}$
					10.10	$1\,{}^{6}A_{1g}$						
$29\ \Gamma_{8g}$	2.926	257	8960	8765.22	26.43	$2\,{}^{6}T_{1g}$	18.31	$1\,{}^{6}A_{1g}$	18.22	$3\,{}^{8}T_{1g}$	15.26	$1\,{}^{6}E_{g}$
					11.47	$1\,{}^{6}T_{1g}$						
$15\ \Gamma_{6g}$	2.926	258	9009	0.53	72.58	$3\,{}^{8}T_{1g}$						
$30\ \Gamma_{8g}$	2.926	257	9060	2295.65	47.76	$3\,{}^{8}T_{1g}$	14.50	$2\,{}^{6}T_{1g}$	12.60	$1\,{}^{6}E_{g}$		
$15\ \Gamma_{7g}$	2.926	257	9128	0.27	46.48	$3\,{}^{8}T_{1g}$	25.17	$1\,{}^{6}E_{g}$	13.23	$2\,{}^{6}T_{1g}$		
$31\ \Gamma_{8g}$	2.924	258	9533	1742.55	27.59	$1\,{}^{6}T_{1g}$	20.71	$1\,{}^{6}T_{2g}$	12.76	$2\,{}^{6}T_{2g}$	10.30	$1\,{}^{6}A_{2g}$
$32\ \Gamma_{8g}$	2.924	258	9630	536.30	27.07	$1\,{}^{6}T_{2g}$	18.79	$1\,{}^{6}T_{1g}$	16.19	$1\,{}^{6}A_{2g}$	11.13	$2\,{}^{6}T_{1g}$
					10.39	$2\,{}^{6}T_{2g}$						
$16\ \Gamma_{6g}$	2.925	257	9749	152.04	52.23	$1\,{}^{6}T_{2g}$						
$16\ \Gamma_{7g}$	2.926	257	10120		66.19	$3\,{}^{8}T_{1g}$	16.48	$1\,{}^{6}A_{1g}$				
$33\ \Gamma_{8g}$	2.926	257	10163	0.02	77.69	$3\,{}^{8}T_{1g}$						
$17\ \Gamma_{7g}$	2.923	257	10415	0.01	38.54	$1\,{}^{6}T_{1g}$	21.70	$2\,{}^{6}E_{g}$	11.60	$3\,{}^{6}T_{1g}$		
$34\ \Gamma_{8g}$	2.924	257	10541	2672.67	34.37	$1\,{}^{6}T_{2g}$	13.70	$2\,{}^{6}T_{1g}$	10.18	$1\,{}^{6}T_{1g}$		
$17\ \Gamma_{6g}$	2.924	257	10656	367.85	28.35	$1\,{}^{6}A_{2g}$	21.78	$2\,{}^{6}T_{2g}$	15.21	$1\,{}^{6}T_{2g}$	13.23	$1\,{}^{6}T_{1g}$
					11.94	$3\,{}^{6}T_{1g}$						
$35\ \Gamma_{8g}$	2.925	258	10694	1418.15	28.11	$1\,{}^{6}T_{1g}$	26.02	$1\,{}^{6}E_{g}$	12.89	$2\,{}^{6}T_{1g}$		
$18\ \Gamma_{7g}$	2.926	257	10730	0.01	39.19	$2\,{}^{6}T_{1g}$	15.99	$1\,{}^{6}A_{1g}$	13.51	$3\,{}^{8}T_{1g}$	10.85	$1\,{}^{6}T_{2g}$
$18\ \Gamma_{6g}$	2.924	257	11326	7.12	36.09	$1\,{}^{6}E_{g}$	24.00	$2\,{}^{6}E_{g}$	13.80	$2\,{}^{6}T_{1g}$	12.99	$2\,{}^{6}T_{2g}$
$36\ \Gamma_{8g}$	2.925	257	11408	0.26	24.00	$2\,{}^{6}T_{1g}$	19.82	$2\,{}^{6}T_{2g}$	19.20	$2\,{}^{6}E_{g}$	10.86	$1\,{}^{6}A_{1g}$
					10.32	$1\,{}^{6}E_{g}$						
$19\ \Gamma_{6g}$	2.924	257	11425	330.41	45.89	$3\,{}^{6}T_{1g}$	19.88	$1\,{}^{6}T_{1g}$	16.58	$2\,{}^{6}T_{2g}$		
$37\ \Gamma_{8g}$	2.924	257	11486	1048.00	37.27	$3\,{}^{6}T_{1g}$	16.04	$1\,{}^{6}T_{2g}$	12.01	$1\,{}^{6}T_{1g}$	11.84	$2\,{}^{6}T_{1g}$
$38\ \Gamma_{8g}$	2.924	257	11680	102.27	46.41	$2\,{}^{6}T_{2g}$	19.57	$2\,{}^{6}E_{g}$				
$19\ \Gamma_{7g}$	2.924	257	11738	0.12	28.63	$3\,{}^{6}T_{1g}$	26.78	$2\,{}^{6}T_{1g}$	20.49	$1\,{}^{6}T_{2g}$	10.54	$1\,{}^{6}T_{1g}$
$39\ \Gamma_{8g}$	2.924	257	11758	731.64	37.07	$1\,{}^{6}T_{2g}$	14.34	$1\,{}^{6}A_{2g}$	13.46	$3\,{}^{6}T_{1g}$	10.44	$2\,{}^{6}E_{g}$
					10.40	$2\,{}^{6}T_{2g}$						
$20\ \Gamma_{7g}$	2.923	257	12085		31.54	$1\,{}^{6}T_{1g}$	31.08	$3\,{}^{6}T_{1g}$	18.73	$2\,{}^{6}T_{2g}$		
$40\ \Gamma_{8g}$	2.925	257	12252	28.50	21.52	$2\,{}^{6}T_{2g}$	20.96	$1\,{}^{6}T_{1g}$	17.96	$2\,{}^{6}E_{g}$	10.15	$1\,{}^{6}T_{2g}$

20 $\mathit{\Gamma}_{6g}$	2.925	257	12335	13.73	27.24	1 $^6T_{2g}$	26.80	2 ^6E_g	16.09	1 ^6E_g	12.82	2 $^6T_{1g}$
					10.58	2 $^6T_{2g}$						
41 $\mathit{\Gamma}_{8g}$	2.924	257	12734	128.95	28.68	1 $^6T_{1g}$	23.65	1 $^6T_{2g}$	14.51	2 $^6T_{2g}$	10.91	1 ^6E_g
21 $\mathit{\Gamma}_{6g}$	2.925	257	12876	2.89	43.61	2 $^6T_{1g}$	28.38	1 ^6E_g	10.13	1 $^6T_{2g}$		
42 $\mathit{\Gamma}_{8g}$	2.925	257	12909	3.60	21.78	1 $^6T_{1g}$	16.58	2 $^6T_{1g}$	16.40	1 $^6T_{2g}$	12.54	3 $^6T_{1g}$
					10.21	2 $^6T_{2g}$						
21 $\mathit{\Gamma}_{7g}$	2.923	257	13223	0.10	32.23	3 $^6T_{1g}$	28.83	2 ^6E_g	14.12	1 $^6T_{1g}$		
43 $\mathit{\Gamma}_{8g}$	2.924	257	13265	849.98	27.02	3 $^6T_{1g}$	17.52	1 ^6E_g	15.28	2 $^6T_{2g}$	14.68	2 ^6E_g
					14.26	2 $^6T_{1g}$						
22 $\mathit{\Gamma}_{6g}$	2.925	258	13329	48.57	34.25	1 $^6T_{2g}$	20.95	1 $^6T_{1g}$	18.03	2 $^6T_{2g}$	15.46	1 $^6A_{2g}$
22 $\mathit{\Gamma}_{7g}$	2.924	257	13347	0.01	27.89	3 $^6T_{1g}$	20.32	2 $^6T_{2g}$	19.38	2 $^6T_{1g}$	17.17	1 $^6A_{1g}$
23 $\mathit{\Gamma}_{7g}$	2.924	257	13902	0.01	26.17	2 ^6E_g	24.48	1 $^6T_{1g}$	16.31	3 $^6T_{1g}$	14.43	1 $^6A_{1g}$
44 $\mathit{\Gamma}_{8g}$	2.924	257	13922	267.47	38.65	2 ^6E_g	21.81	2 $^6T_{2g}$	15.46	3 $^6T_{1g}$		
45 $\mathit{\Gamma}_{8g}$	2.924	257	14018	280.07	24.82	2 $^6T_{1g}$	14.89	1 ^6E_g	13.68	3 $^6T_{1g}$	11.58	1 $^6T_{1g}$
23 $\mathit{\Gamma}_{6g}$	2.924	257	14298	18.06	40.08	2 $^6T_{2g}$	26.31	3 $^6T_{1g}$	11.68	2 $^6T_{1g}$		
46 $\mathit{\Gamma}_{8g}$	2.924	257	14359	1.99	26.67	2 $^6T_{1g}$	23.73	2 $^6T_{2g}$	22.26	1 ^6E_g		
47 $\mathit{\Gamma}_{8g}$	2.924	257	14728	0.34	26.29	3 $^6T_{1g}$	19.98	2 $^6T_{2g}$	14.50	2 $^6T_{1g}$	12.79	2 ^6E_g
24 $\mathit{\Gamma}_{7g}$	2.924	257	15076	0.01	46.24	2 $^6T_{1g}$	29.23	1 ^6E_g				
24 $\mathit{\Gamma}_{6g}$	2.923	257	15269	13.06	63.39	2 $^6T_{2g}$	26.39	2 ^6E_g				
48 $\mathit{\Gamma}_{8g}$	2.922	257	15396	5.13	47.79	3 $^6T_{1g}$	24.41	2 $^6T_{2g}$	12.99	2 ^6E_g		
25 $\mathit{\Gamma}_{7g}$	2.922	257	15808		38.18	2 $^6T_{2g}$	38.16	3 $^6T_{1g}$				
49 $\mathit{\Gamma}_{8g}$	2.922	256	16446	0.45	73.91	3 $^6T_{1g}$	15.39	2 $^6T_{2g}$				

[a] The analyses of the wave functions have been done at $d_{\mathrm{Tb-S}}$= 2.700 Å ($4f^8$), 2.950 Å ($4f^85d^1$), 2.950 Å ($4f^9$).
[b] C.f. Table IV.
[c] C.f. Table V.

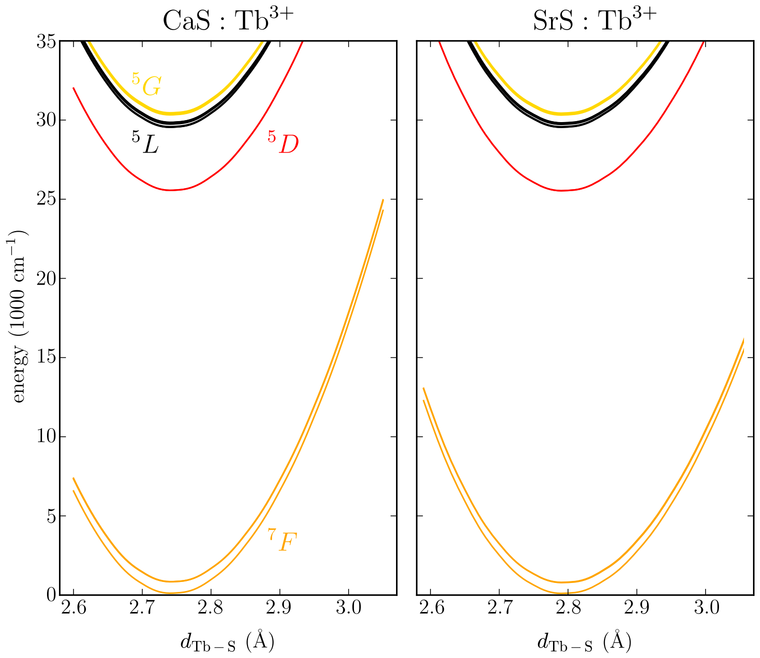

Supplementary Figure 10. Potential energy curves at spin-orbit-free SA-RASSCF level for states of the 4f^8 configuration of the octahedral Tb^{3+} impurity in the alkaline earth sulfides CaS and SrS. The color of the curves denotes the main L value of the associated ^{2S+1}L term.

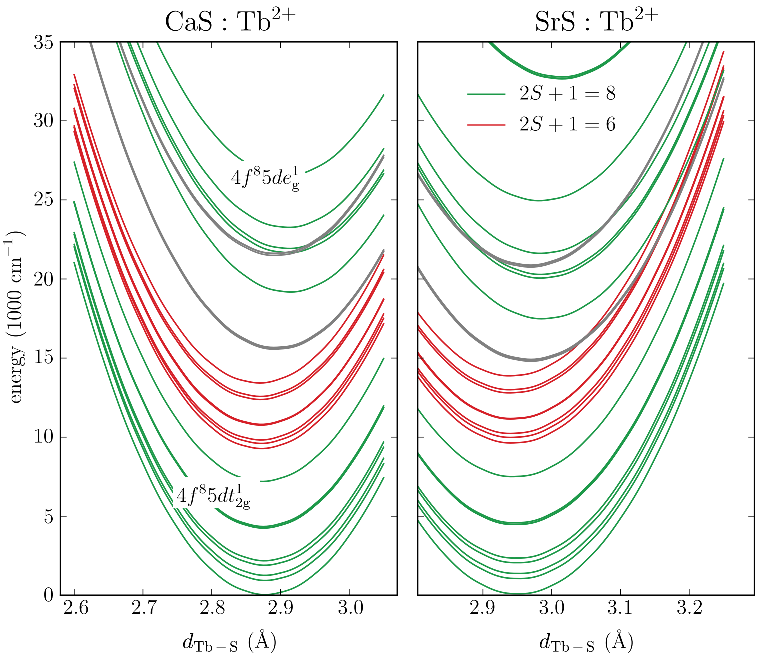

Supplementary Figure 11. Potential energy curves at spin-orbit-free SA-RASSCF level for the Tb^{2+} impurity in the alkaline earth sulfides CaS and SrS. Green and red curves originate from the states with 4f^8(5d,6s)^1 configurations, indicating spin-octet and spin-sextet levels respectively. The grey curves show the levels that correspond to the 4f^9 configuration, from low to high energy the ^6H, ^6F and ^6P terms.

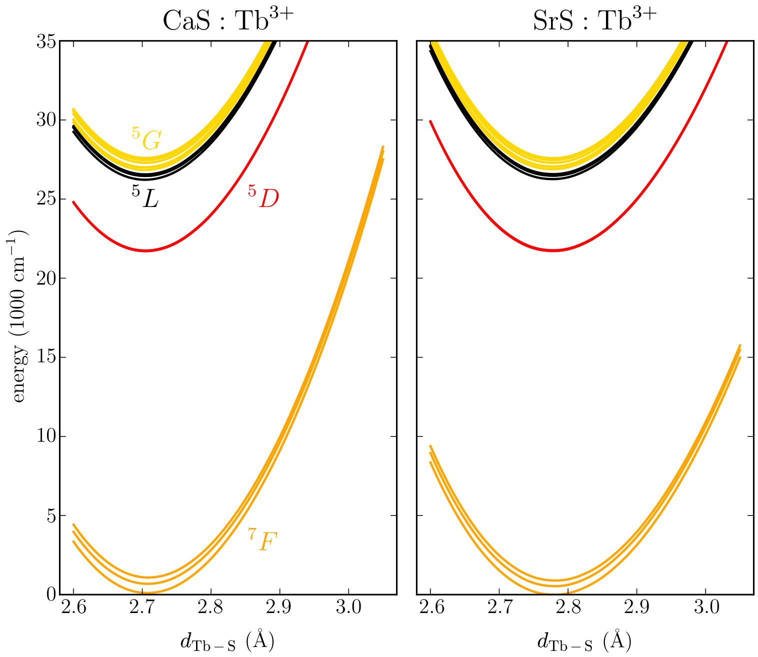

Supplementary Figure 12. Potential energy curves at spin-orbit-free MS-RASPT2 level for states of the $4f^8$ configuration of the octahedral Tb^{3+} impurity in the alkaline earth sulfides CaS and SrS. The color of the curves denotes the main L value of the associated ^{2S+1}L term.

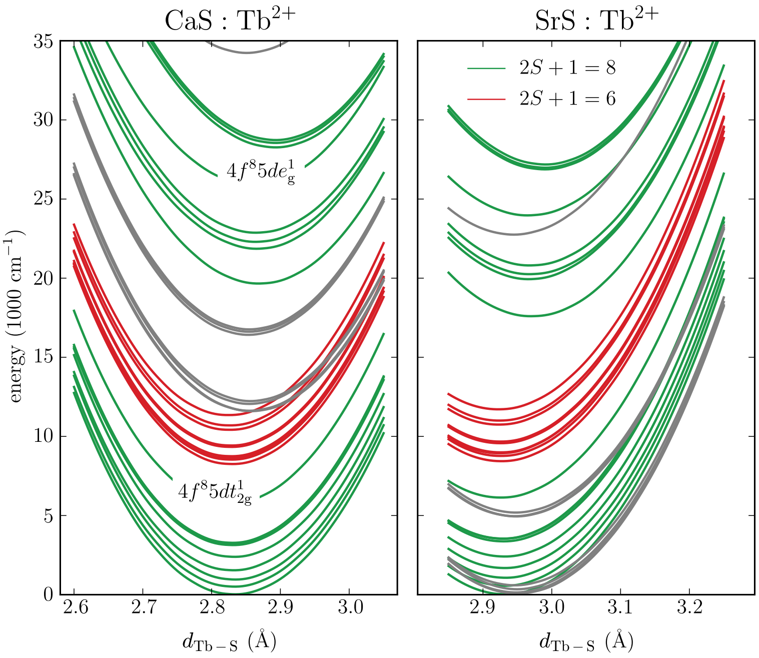

Supplementary Figure 13. Potential energy curves at spin-orbit-free MS-RASPT2 level for the Tb^{2+} impurity in the alkaline earth sulfides CaS and SrS. Green and red curves originate from the states with $4f^8(5d,6s)^1$ configurations, indicating spin-octet and spin-sextet levels respectively. The grey curves show the levels that correspond to the $4f^9$ configuration, from low to high energy the ^6H, ^6F and ^6P terms.

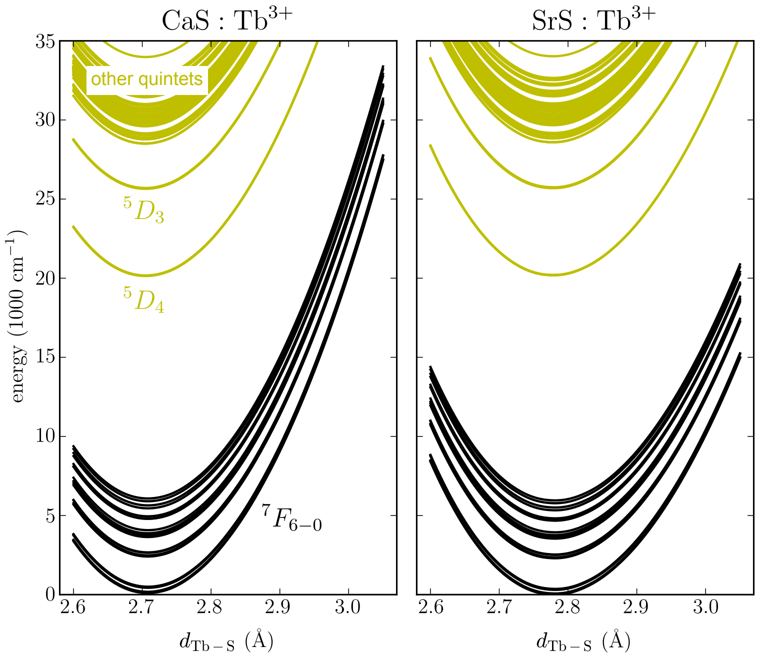

Supplementary Figure 14. Potential energy curves after spin-orbit coupling (at RASSI-SO level) for states of the $4f^8$ configuration of the octahedral Tb^{3+} impurity in the alkaline earth sulfides CaS and SrS. The black curves correspond to the ^{7}F multiplets, the gold curves to the lowest-lying multiplets for which $2S + 1 = 5$.

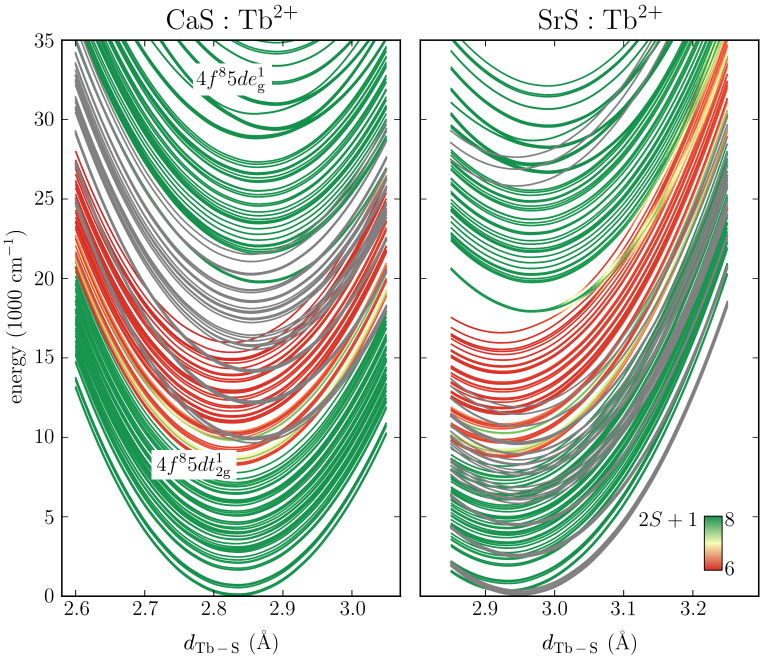

Supplementary Figure 15. Potential energy curves after spin-orbit coupling (at RASSI-SO level) for the Tb^{2+} impurity in the alkaline earth sulfides CaS and SrS. The colored curves originate from the $4f^8(5d, 6s)^1$ configurations, whereby the color represents the spin-character of the eigenstates, ranging from green for pure spin-octets to red for pure spin-sextets. The grey curves show the levels that correspond to the $4f^9$ configuration.
